# Structural, mechanistic and functional insight into gliotoxin *bis*-thiomethylation in *Aspergillus fumigatus*

**DOI:** 10.1098/rsob.160292

**Published:** 2017-02-08

**Authors:** Stephen K. Dolan, Tobias Bock, Vanessa Hering, Rebecca A. Owens, Gary W. Jones, Wulf Blankenfeldt, Sean Doyle

**Affiliations:** 1 Department of Biology, Maynooth University, Maynooth, Co. Kildare, Ireland; 2 Helmholtz Centre for Infection Research, Structure and Function of Proteins, Inhoffenstraße 7, 38124 Braunschweig, Germany; 3 Institute of Biochemistry, Biotechnology and Bioinformatics, Technische Universität Braunschweig, Spielmannstrasse 7, 38106 Braunschweig, Germany

**Keywords:** methyltransferase, NRPS, quantitative proteomics, *Aspergillus*

## Abstract

Gliotoxin is an epipolythiodioxopiperazine (ETP) class toxin, contains a disulfide bridge that mediates its toxic effects via redox cycling and is produced by the opportunistic fungal pathogen *Aspergillus fumigatus*. Self-resistance against gliotoxin is effected by the gliotoxin oxidase GliT, and attenuation of gliotoxin biosynthesis is catalysed by gliotoxin *S*-methyltransferase GtmA. Here we describe the X-ray crystal structures of GtmA-apo (1.66 Å), GtmA complexed to *S*-adenosylhomocysteine (1.33 Å) and GtmA complexed to *S*-adenosylmethionine (2.28 Å), providing mechanistic insights into this important biotransformation. We further reveal that simultaneous elimination of the ability of *A. fumigatus* to dissipate highly reactive dithiol gliotoxin, via deletion of GliT and GtmA, results in the most significant hypersensitivity to exogenous gliotoxin observed to date. Indeed, quantitative proteomic analysis of Δ*gliT*::Δ*gtmA* reveals an uncontrolled over-activation of the *gli*-cluster upon gliotoxin exposure. The data presented herein reveal, for the first time, the extreme risk associated with intracellular dithiol gliotoxin biosynthesis—in the absence of an efficient dismutation capacity. Significantly, a previously concealed protective role for GtmA and functionality of ETP *bis*-thiomethylation as an ancestral protection strategy against dithiol compounds is now evident.

## 1. Introduction

Ascomycetes constitute the largest phylum of the fungal kingdom and produce a copious array of natural products. Although many of these compounds are known as clinically important drugs or industrial chemicals, several natural products are potent toxins that pose substantial threats to human food supplies and health [1]. Production of these natural products requires a carefully orchestrated system to balance biosynthesis while avoiding self-harm from endogenous accumulation of toxic natural product precursors [2].

**Figure 1.**
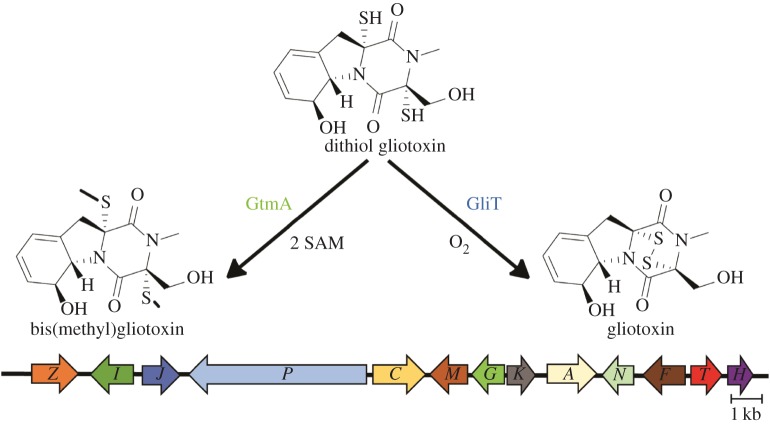
Conversion of gliotoxin between the reduced (dithiol gliotoxin), oxidized (gliotoxin) and bisthiomethyl forms. In *A. fumigatus*, the *gli*-cluster that encodes gliotoxin biosynthesis consists of 13 genes (in colour and labelled with their last letter) and is located on chromosome 6 [4–6]. *gtmA* is encoded outside the cluster and is on chromosome 2.

Epipolythiodioxopiperazine (ETP) alkaloids are toxic natural products characterized by a unique bridged disulfide or polysulfide dioxopiperazine ring. Gliotoxin, produced by the opportunistic fungus *Aspergillus fumigatus*, is the prototypic member of this large family [3]. As with other ETPs, gliotoxin is produced through a sequential series of enzymatic steps which are organized into a coordinated biosynthetic gene cluster (figure 1). Although the mechanism of action for the cytotoxicity of gliotoxin has not been fully elucidated, two primary activities have been described: the generation of reactive oxygen species (ROS) through oxidation of the disulfide bridge and mixed disulfide formation. The stereochemically complex core of ETPs, coupled with their potent biological activities, make these compounds an attractive target for drug leads [7].

Resistance genes are required to allow a toxin producer to grow in the presence of its own metabolic weaponry, and ETPs are no exception to this rule [8]. Indeed, one of the genes in the gliotoxin biosynthetic gene cluster in *A. fumigatus*, *gliT*, gliotoxin oxidase, is required for self-protection against the toxin [4,9]. Deletion of this gene renders *A. fumigatus* sensitive to exogenous gliotoxin. Enzyme-catalysed epidisulfide formation appears to be restricted to ETP producers, despite the fact that heterologous expression of GliT in *A. nidulans* or *Saccharomyces cerevisiae* provided effective cross-species resistance to gliotoxin [4].

Several fungi have been shown to carry out an irreversible enzymatic *bis*-thiomethylation of these disulfide-containing metabolites. These *S*-methylated ETP derivatives have a significantly dulled bioactivity. *S*-methylation of dithiol metabolites also extends to bacteria, and *Streptomyces clavuligerus* has been shown to produce a *bis*-thiomethylated derivative of the dithiolpyrrolone antibiotic holomycin [10]. This thiomethylation mechanism has been posited as an additional or backup strategy to disulfide bridge closure for self-protection during holomycin biosynthesis. It has been proposed that *S*-methylation of biosynthetic intermediates or possibly shunt metabolites protect cellular components against these reactive species [10,11].

We and others have shown that *A. fumigatus* effects dithiol gliotoxin *S*-methylation via a methyltransferase, gliotoxin thiomethyltransferase A (GtmA or TmtA) located outside the gliotoxin gene cluster [12,13]. Deletion of *gtmA* resulted in no additional sensitivity to exogenous gliotoxin [12], but completely abrogated *bis*(methyl)gliotoxin (BmGT) production in this organism. This led us to conclude that GtmA is not primarily involved in the detoxification of gliotoxin or related biosynthetic intermediates [14] as has been previously proposed [10]. We subsequently demonstrated that GtmA-mediated *bis*-thiomethylation of gliotoxin by *A. fumigatus* regulates the production of this toxin in *A. fumigatus* by disrupting a positive feedback loop which normally potentiates gliotoxin biosynthesis [12].

Are non-ETP producers capable of protecting themselves against these potent natural products? Recently, it was shown that disruption of the uncharacterized methyltransferase *MT-II* in *A. niger* resulted in increased sensitivity to exogenous gliotoxin. Like GtmA, recombinantly expressed MT-II was shown to sequentially *bis*-thiomethylate dithiol gliotoxin, forming monomethylgliotoxin (MmGT) and then BmGT [15]. MT-II is an orthologue of GtmA (53% sequence identity), despite the fact that this organism does not produce gliotoxin. This suggested that *bis*-thiomethylation may have an ancestral role in protecting organisms against dithiol-containing toxins in selected filamentous fungi, such as *A. niger* and *A. nidulans* [13,15]. However, it appears that ETP self-protection in producer organisms is dominated by reversible enzyme-catalysed epidisulfide formation, whereas the permanent, metabolically expensive mechanism of ETP *S*-methylation has become specialized to regulate ETP production in filamentous fungi [12].

We report crystal structures of apo-, SAM- and SAH-bound GtmA, offering further mechanistic insights into this elusive biochemical transformation. The presence of a dominant gliotoxin self-protection mechanism in *A. fumigatus* (GliT) hinders our understanding of the contribution of ETP *bis*-thiomethylation to self-protection. Here, we demonstrate that a double deletion mutant of *gliT* and *gtmA* struggles to grow in the presence of low amounts of exogenous gliotoxin. Using quantitative proteomics and metabolomics, we also demonstrate that the inability to derivatize gliotoxin results in an unhindered upregulation of the gliotoxin biosynthetic pathway in this organism—leading to hypersensitivity.

## 2. Results

### 2.1. Comparison of apo-, SAM- and SAH-complexed GtmA

In order to obtain detailed insights into the enzymatic mechanism of GtmA-catalysed methyltransfer, we crystallized GtmA in the cofactor-free form and in complex with SAM and SAH, and refined the structures at 1.66 Å, 2.28 Å and 1.33 Å resolution with *R*-factors of *R*_apo_ = 16.8, *R*_SAM_ = 19.2, *R*_SAH_ = 13.9, and *R*_free_-values of *R*_freeapo_ = 18.6, *R*_freeSAM_ = 23.4, *R*_freeSAM_ = 16.6, respectively (electronic supplementary material, table S1 and S2). Despite multiple attempts, we were unable to obtain cocrystals of GtmA with either gliotoxin or BmGT bound. The apo form crystallized in space group P6_2_ with one chain in the asymmetric unit, whereas the complexes appeared in two different crystal forms belonging to space group P2_1_ with two monomers as the asymmetric unit in both cases. According to PDBePISA [16], the SAH-complexed GtmA forms a stable dimer, as indicated by a buried surface area of 3450 Å^2^ of 22 640 Å^2^ total surface area and a solvation free energy gain of −81.3 kcal mol^−1^. However, although many natural product methyltransferases are dimeric [17], GtmA appeared to be monomeric during size exclusion chromatography (electronic supplementary material, figure S1). Furthermore, the dimeric arrangement of the SAH complex was not observed in the apo- or SAM-complexed structure, where dimeric arrangements are established via different interfaces (electronic supplementary material, figure S1). Therefore, the dimer found in the SAH complex is probably a crystallization artefact. Electronic supplementary material, figure S2 shows electron densities of SAM and SAH.

**Figure 2.**
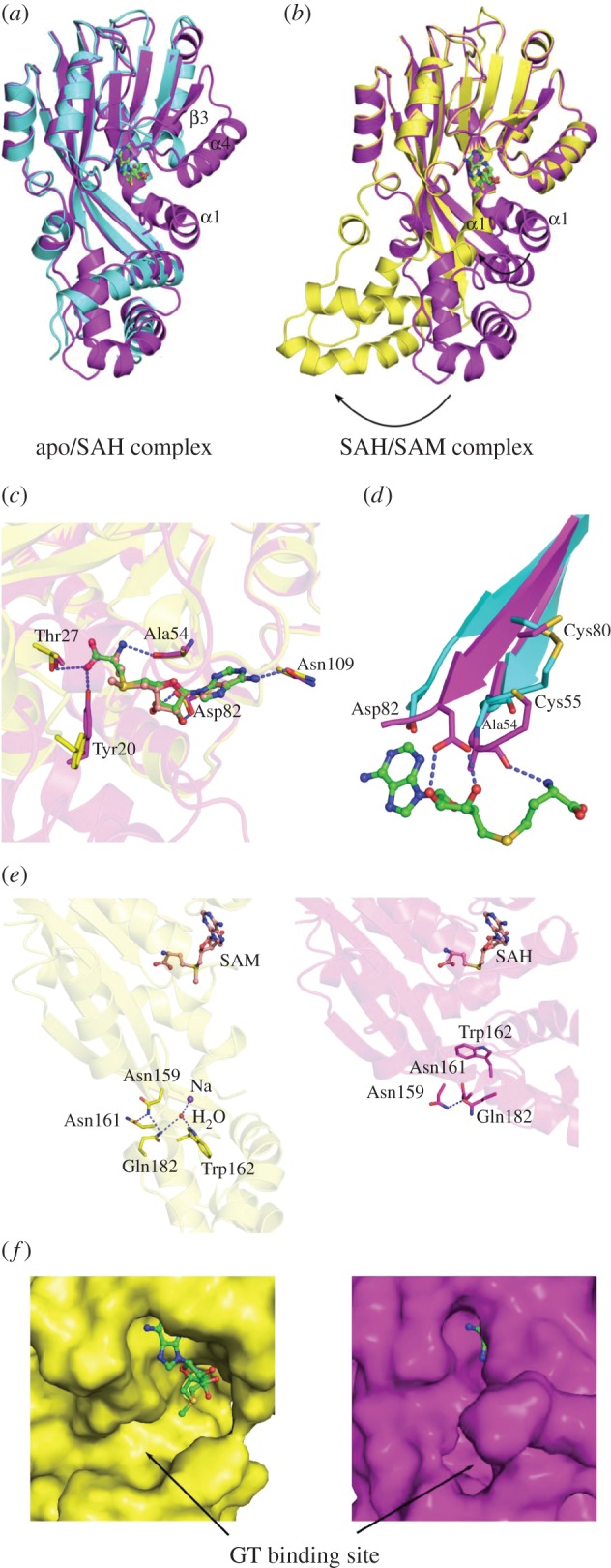
Comparison of apo-GtmA and the SAH/SAM complexes. (*a*) apo- and SAH-bound GtmA. The missing secondary structure elements of apo GtmA are labelled. (*b*) SAH- and SAM-bound complex. The dramatic movement of the lower domain and helix α1 is indicated by arrows. (*c*) Co-factor binding: comparison of SAH (magenta) and SAM (yellow) binding mode. Thr27, Ala54, Asp82 and Asn109 are similar in position, only Tyr20 is flipped out in the SAM structure owing to the movement of the lower domain and helix α1. (*d*) Comparison of apo- (cyan) and SAH-complexed (magenta) GtmA. The disulfide bridge found between Cys55 and Cys80 of the apo structure has to be reduced to allow SAH/SAM binding. (*e*) Asn159 is involved in a hydrogen bonding network with Asn161, Glu182, a Na^+^ cation, a water molecule and Trp162. (*f*) Disruption of the GT binding pocket in the SAM complex. The SAH complex is coloured magenta and the SAM complex yellow. Also see electronic supplementary material, figure S5.

GtmA consists of two domains, a larger ‘upper’ domain containing a Rossmann-fold involved in binding the co-substrate SAM and a smaller ‘lower’ domain that changes its relative position to the upper domain in the three crystal structures described here (figure 2*a,b*). apo-GtmA seems to be highly flexible, as indicated by the higher *B*-factors. The flexibility is more pronounced in the lower domain, which is also evident by the finding that almost no ordered water molecules can be observed in the electron density of this region. The apo structure adopts a similar conformation as the SAH complex; however, parts of the upper domain (the first 22 residues of the N-terminus including parts of α1 and residues 86–114, including the complete secondary structure elements β3 and α4), are not visible in the electron density (figure 2*a*). On the other hand, our SAH- and SAM-bound structures show some striking differences. Whereas the upper domain is almost identical, the lower domain and helix α1 are twisted by 87° [18] in the SAM-bound structure. This movement is mediated by the three linker strands β5–7 (figure 2*a*), and even though the lower domain is involved in crystal contacts, its movement does not seem to be caused by crystallization because it affects both independent monomers in the asymmetric unit to the same extent and was observed only when crystals were grown in the presence of SAM (electronic supplementary material, figure S1). We therefore hypothesize that this conformation represents an intermediate state of GtmA during the methyltransferase reaction cycle. In agreement with Duell *et al*. [19], who have recently published the structure of a GtmA–SAH complex in the same crystal form as described here, the cofactor binding site of GtmA is located in the upper part of a cleft between the two domains built by helix α1, sheet β2 and loop regions connecting β1–α2 and β3–β4 (figure 2*a*). The carboxylate group of SAH is hydrogen bonded to Thr27 and Tyr20, residues located in helix α1. However, in the SAM-bound structure, Tyr20 is flipped out of the binding site. The adenyl component of the cofactor is bound to Asn109, the amino group interacts with the backbone carbonyl atom of Ala54, and the ribose hydroxyl moiety is fixed by Asp82 (figure 2*c,d*).

Interestingly, the two important residues Ala54 and Asp82 are part of β-strands β1 and β2, two regions connected by a disulfide bridge formed by Cys55 and Cys80 in the apo structure (figure 2*c,d*). This disulfide bridge appears to be reduced in the cofactor-bound structures, leading to a movement of the two β-strands towards the cofactor. This motion is a prerequisite to provide the corresponding interaction partners for the binding of the co-substrate SAM (figure 2*b*). We investigated the possible relevance of the disulfide bridge by carrying out microscale thermophoresis (MST) to determine the affinity of GtmA for SAM under reducing and oxidizing conditions; however, the affinity of GtmA for SAM was similar in both cases (electronic supplementary material, figure S3).

**Figure 3.**
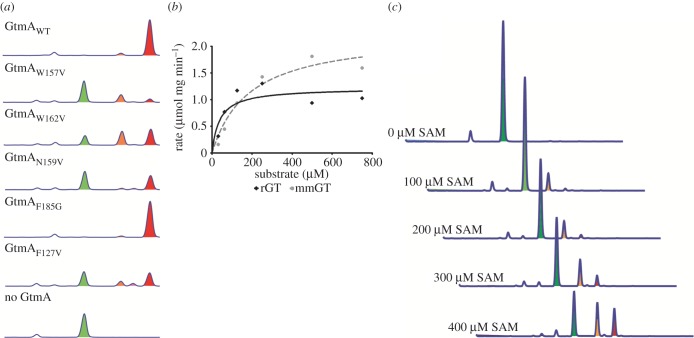
(*a*) RP-HPLC chromatograms of GtmA wild-type, GtmA W157V, GtmA W162V, GtmA N159V, GtmA F185G, GtmA F127V and a ‘no GtmA’ control incubated with dithiol gliotoxin and SAM. Dithiol gliotoxin (green), mono(methylthio)gliotoxin (orange), *bis*(methyl)gliotoxin (red). (*b*) GtmA K_m_ determination for dithiol gliotoxin (rGT; 38.62 µM) was almost fivefold lower than that for purified MmGT (184.5 µM). (*c*) GtmA-mediated *bis*-thiomethylation occurs sequentially. The addition of increasing amounts of SAM (100–400 µM) results in the increased formation of *bis*(methyl)gliotoxin (red) compared with monomethylgliotoxin (orange). Dithiol gliotoxin is shown in green.

A sequence alignment of *A*. *fumigatus* GtmA with the *A. niger* GtmA homologue MT-II indicated residues that have a high degree of conservation [20]. Considering both these methyltransferases catalyse the same reaction, residues which are essential for controlling substrate specificity or catalysis are likely to be conserved. In combination with the crystallographic data shown in figure 2, the five residues W157V, W162V, N159V, F185G and F127V (electronic supplementary material, figure S4) were selected for mutagenesis. These residues are identical in *A*. *fumigatus* GtmA and *A. niger* MT-II (electronic supplementary material, figure S6). As shown in figure 3*a*, methyltransferase activity of the GtmA mutants was monitored by RP-HPLC. Wild-type GtmA converted all dithiol gliotoxin in the reaction (green) to BmGT (red). We found that the Asn159Val mutant generated only low levels of MmGT and BmGT compared with the wild-type enzyme, which might be caused by a disturbed release of the monomethylated form. Asn159 is located at the surface of the apo- and SAH-complexed GtmA, and did not seem to interact with residues putatively involved in dithiol gliotoxin binding or methyltransfer. However, in the SAM complex, it is part of a hydrogen bonding network with Asn161, Gln182, a sodium ion, a water molecule and Trp162 (figure 2*c*). Trp162 itself was shown to be crucial for efficient catalysis (figure 3*a*). This network of hydrogen bonds only appears in the SAM complex and emerges from the large structural movement of the lower domain. Compared with the closed apo- and the SAH-bound structures, this complex is elongated and open, such that the cofactor binding pocket appears accessible (figure 2*e,f*) and the putative dithiol gliotoxin binding site predicted by Duell *et al*. [19] by molecular docking (comprising W162, Y20, F11, T27, M10, F185) is disrupted (figure 2*c*; electronic supplementary material, figure S5). Therefore, we propose that the SAM complex observed in our data represents the state before dithiol gliotoxin binds. Further, we hypothesize that the lower domain functions as a carrier that locates the substrate to its proper position and thereby closes the active site. After the first methyltransfer, the lower domain moves back to the position found in the SAM complex, SAH is exchanged for SAM and the second methylation can occur in the closed state. This conformational change is impaired in the Asn159Val variant, leading to a disturbed release mechanism and to the production of lower levels of MmGT and BmGT.

Trp157Val and Trp162Val had a significant effect on the activity of the enzyme as these residues have been proposed to stabilize the diketopiperazine core of dithiol gliotoxin [19]. The conversion of dithiol gliotoxin to BmGT is significantly diminished when the Trp157Val mutant is used to effect catalysis (figure 3*a*). Almost no BmGT is generated by this enzyme, and only low quantities of MmGT are apparent. Phe127Val also resulted in a significant decrease in GtmA activity.

### 2.2. Mechanistic insights into GtmA activity

Considering that GtmA is the first methyltransferase identified with dual ETP *S*-methylation activity, mechanistic and structural insight into the process of GtmA substrate binding and the methylation sequence is of considerable interest. The GtmA *K*_m_ determined for dithiol gliotoxin (38.62 µM) was almost fivefold lower than that for purified MmGT (184.5 µM; figure 3*b*), which suggests the former is the preferred substrate for the enzyme. Moreover, time-course analysis of GtmA activity over 60 min revealed that almost 50% of added dithiol gliotoxin is converted to MmGT by 20 min, prior to commencement of detectable BmGT formation (electronic supplementary material, figure S7). As shown in figure 3*c*, the conversion of dithiol gliotoxin to MmGT/BmGT by GtmA was also monitored under SAM-limiting conditions. MmGT is detected as the primary reaction product when SAM is limiting in the methyltransfer reaction (100–200 µM), and BmGT appears to only be generated following the conversion of dithiol gliotoxin to MmGT. Adding additional SAM to the SAM-limited reactions resulted in full conversion of the MmGT to BmGT, thus proving that GtmA can bind to free MmGT (electronic supplementary material, figure S7). This suggests that after the first *S*-methylation, MmGT leaves the GtmA complex and is then taken up for methylation on the opposite thiol by a second GtmA molecule with SAM bound. Based on these data, it appears that GtmA has higher affinity for dithiol gliotoxin than for MmGT, resulting in the preferential modification of dithiol gliotoxin to MmGT before the *S*-methylation of MmGT in the second position. The fact that GtmA does not appear to hold its substrate bound for a second methylation suggests that GtmA is not a processive enzyme, unlike previously characterized natural product methyltransferases which mediate consecutive methyltransfers [21].

### 2.3. GtmA is not subject to SAH-mediated feedback inhibition

The majority of SAM-dependent methyltransferases are known to be inhibited by SAH, the methyl-depleted version of SAM [22]. Recombinant GtmA was pre-incubated with SAH for 30 min (400 µM) to determine if this metabolite had an inhibitory effect on *bis*-thiomethylation activity. No significant difference in BmGT production was noted in samples containing SAH or control samples as detected by RP-HPLC (electronic supplementary material, figure S8). These results suggest that GtmA was resistant to SAH-mediated feedback inhibition, indicating a low affinity of GtmA towards SAH. This is in line with the proposal that SAH has to be released so that GtmA can undertake a new methyltransfer reaction.

### 2.4. The *Aspergillus fumigatus* double mutants Δ*gliT::*Δ*gtmA and* Δ*gliA::*Δ*gtmA* exhibit decreased growth upon gliotoxin exposure

**Figure 4.**
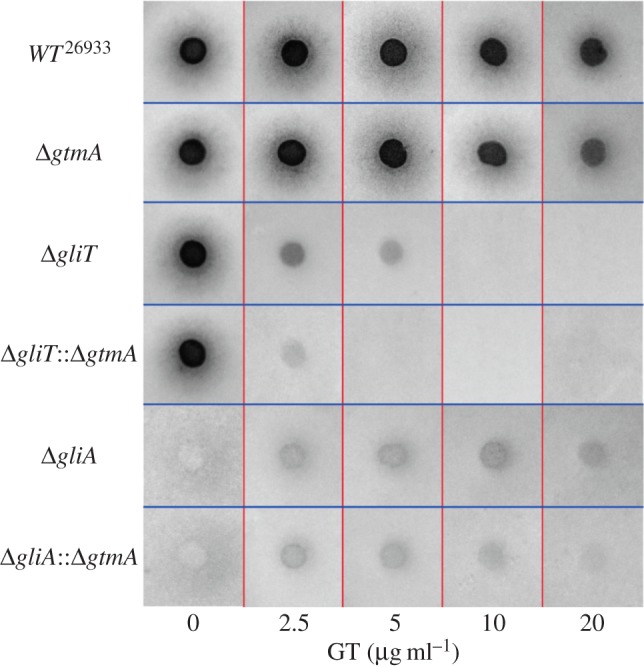
(*a*) Deletion of *gtmA* does not result in gliotoxin sensitivity when compared with *A. fumigatus* wild-type. Deletion of *gtmA* in the gliotoxin-sensitive strains Δ*gliT* and Δ*gliA* resulted in double mutants with an increased sensitivity to gliotoxin exposure. *Aspergillus fumigatus* Δ*gliT*::Δ*gtmA* was shown to be particularly sensitive to exogenous gliotoxin whereby the radial growth of this mutant was inhibited at 2.5 µg ml^−1^ gliotoxin.

Expression of the *A. fumigatus* dithiol oxidase gene *gliT* is required for self-protection against the gliotoxin [4,9]. Similarly, expression of *gliA*, a major facility superfamily transporter encoded within the *gli*-cluster, is also required for tolerance to gliotoxin [23]. Expression of *gtmA* was disrupted in the gliotoxin-sensitive ATCC26933 backgrounds Δ*gliT* and Δ*gliA* (resulting in Δ*gliT*::Δ*gtmA* and Δ*gliA*::Δ*gtmA*) for comparison with the Δ*gtmA* mutant (electronic supplementary material, figure S9–S11). Plate assays were performed on Czapek Dox agar containing gliotoxin (0–20 µg ml^−1^) to determine the response of both Δ*gliT*::Δ*gtmA* and Δ*gliA*::Δ*gtmA* to exogenous gliotoxin exposure. Radial growth was observed at 24 h time points, and the plates were imaged at 72 h (figure 4). Upon exposure to gliotoxin, both *A. fumigatus* Δ*gliT*::Δ*gtmA* (*p* < 0.0001) and Δ*gliA*::Δ*gtmA* (*p* < 0.0021) exhibited significantly decreased growth in comparison with their single-deletion counterparts. Specifically, Δ*gliT*::Δ*gtmA* was extremely sensitive to exogenous gliotoxin exposure.

### 2.5. Deletion of *gtmA* in Δ*gliT* relieves cellular SAM depletion following gliotoxin exposure

**Figure 5.**
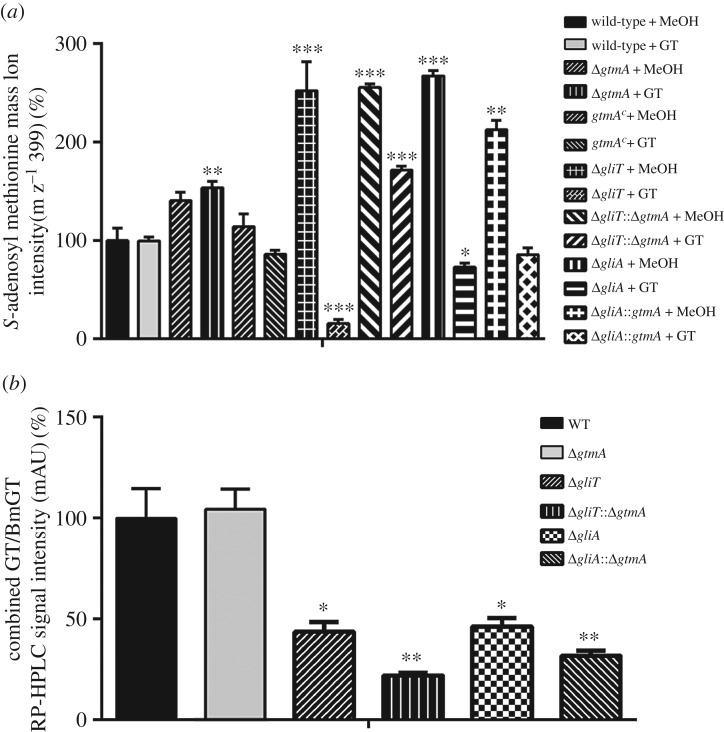
(*a*) SAM detection in *A. fumigatus* wild-type and selected mutants after 21 h growth in Czapek Dox liquid medium followed by 3 h exposure to control (MeOH) or gliotoxin exposure (5 µg ml^−1^ final). Compared with the wild-type, significantly higher cellular SAM was detectable in Δ*gliT* (*p* = 0.0093), Δ*gliA* (*p* = 0.0003), Δ*gliT*::Δ*gtmA* (*p* = 0.0003) and Δ*gliA*::Δ*gtmA* (*p* = 0.0021). Gliotoxin exposure results in highly significant SAM depletion in Δ*gliT* (*p* = 0.0001). This SAM depletion does not occur in the Δ*gliT:*:Δ*gtmA* mutant. SAM levels were also significantly reduced in Δ*gliA* upon gliotoxin exposure (*p* = 0.0100). In comparison, this reduction was not significant in the Δ*gliA*::Δ*gtmA* double mutant. SAM depletion did not occur in Δ*gtmA* to the same degree as in the wild-type or complemented strain (*p* = 0.0021). (*b*) Exposure of wild-type and mutant strains to 2.5 µg ml^−1^ gliotoxin for 3 h and quantification of the remaining GT/BmGT in the organically extracted supernatants. The bars represent the combined intensity of GT/BmGT signal on the RP-HPLC 280 nm chromatogram. *Aspergillus fumigatus* Δ*gliT*::Δ*gtmA* has significantly lower extracellular GT/BmGT than Δ*gliT* after 3 h (*p* = 0.0118).

Considering that we previously suggested a role for SAM:SAH deregulation in the increased sensitivity of Δ*gliT* to gliotoxin [24], the cellular levels of SAM were evaluated in Δ*gliT*::Δ*gtmA* upon gliotoxin exposure compared with a solvent control. As revealed in figure 5*a*, the comparison of cellular SAM levels across the *gli-*cluster deletion strains revealed a close link between GtmA activity and cellular SAM availability following gliotoxin exposure. Δ*gtmA* exhibited significantly (*p* = 0.0021) higher cellular SAM levels compared with the wild-type strain after gliotoxin exposure, which is probably due to the absence of *bis*-thiomethylation. SAM levels were restored to those of wild-type in *gtmA*^C^. Notably, the severe SAM depletion which was identified in Δ*gliT* following gliotoxin exposure (*p* = 0.0001) was alleviated in Δ*gliT*::Δ*gtmA*. *Aspergillus fumigatus* Δ*gliA*::Δ*gtmA* was also shown to retain higher levels of cellular SAM than Δ*gliA* following gliotoxin exposure; however, this was not as drastic as the changes seen in Δ*gliT* compared with Δ*gliT*::Δ*gtmA*. The corresponding LC–MS chromatograms are shown in electronic supplementary material, figure S12. These results directly link GtmA-mediated *bis*-thiomethylation activity and SAM utilization in *A. fumigatus* and strongly suggest that the sensitivity of Δ*gliT*::Δ*gtmA* to gliotoxin was not primarily due to the deregulation of SAM:SAH.

### 2.6. Label-free quantitative proteomic analysis of *Aspergillus fumigatus* wild-type, Δ*gtmA*, Δ*gliT* and Δ*gliT*::Δ*gtmA* exposed to gliotoxin

Gliotoxin sensitivity assays (figure 4) demonstrated that Δ*gliT*::Δ*gtmA* is extremely sensitive to exogenous gliotoxin but, unlike *A. fumigatus* Δ*gliT*, this mutant did not undergo SAM depletion following gliotoxin exposure (figure 5*a*). In order to examine the extreme gliotoxin sensitivity of Δ*gliT*::Δ*gtmA* compared with the response of the single mutants Δ*gliT* and Δ*gtmA*, the proteomic signature of *A. fumigatus* wild-type and these mutants in response to either gliotoxin (2.5 µg ml^−1^, 3 h) or a solvent control was characterized. As shown in table 1, between 2375 and 2512 proteins were detected (triplicate) across all samples. This allowed us to directly compare the individual response of each strain to gliotoxin exposure. As shown in table 1, the number of proteins deregulated in abundance upon gliotoxin exposure correlated with the sensitivity of these strains to the toxin. *Aspergillus fumigatus* wild-type and Δ*gtmA*, which are not inhibited by exposure to 2.5 µg ml^−1^ gliotoxin, showed a total of 168 (wild-type) and 147 (Δ*gtmA*) proteins altered in abundance. *Aspergillus fumigatus* Δ*gliT*, which is moderately sensitive to 2.5 µg ml^−1^ gliotoxin, showed a total of 416 proteins altered in abundance. The highly gliotoxin-sensitive Δ*gliT*::Δ*gtmA* showed a total of 548 proteins significantly altered in abundance upon gliotoxin exposure (electronic supplementary material, table S3 and figures S13–S18). This result highlighted that a drastic proteomic remodelling occurs in Δ*gliT*::Δ*gtmA* in response to this toxin.

**Table 1.** Proteomic analysis of selected *A. fumigatus* mutants exposed to gliotoxin (2.5 µg ml^−1^) or a solvent (MeOH) control. The quantity of proteins deregulated in abundance upon gliotoxin exposure correlates with the sensitivity of these mutants to gliotoxin.

condition	total proteins detected	higher abundance (+)	uniquely detected	lower abundance (−)	below detection limit
Wild-type GT versus MeOH	2512	79	4	45	40
Δ*gtmA* GT versus MeOH	2505	101	5	16	25
Δ*gliT* GT versus MeOH	2472	211	6	157	42
Δ*gliT::*Δ*gtmA* GT versus MeOH	2449	274	9	231	34
Δ*gliT* versus Δ*gliT::*Δ*gtmA* GT	2375	29	8	18	9
Δ*gliT* versus Δ*gliT::*Δ*gtmA* MeOH	2487	1	4	6	13

The Δ*gliT* versus Δ*gliT*::Δ*gtmA* LFQ proteomic analysis samples exposed to gliotoxin were directly compared in order to elucidate the reason behind the enhanced gliotoxin sensitivity of Δ*gliT*::Δ*gtmA*. Twenty-four proteins were uniquely detected in the Δ*gliT*::Δ*gtmA* under gliotoxin exposure, and 37 proteins were significantly more abundant in this mutant. Twelve proteins were uniquely detected in Δ*gliT*, and 22 proteins were shown to be significantly more abundant in this mutant compared with Δ*gliT*::Δ*gtmA* (electronic supplementary material, table S3 and figures S13–S18).

As shown in table 2, the proteins most significantly increased in abundance in Δ*gliT*::Δ*gtmA* compared with either Δ*gliT* or Δ*gtmA* upon gliotoxin exposure are encoded by the gliotoxin biosynthetic cluster. Several proteins detected as significantly less abundant in Δ*gliT*::Δ*gtmA* were shown to be associated with the ribosome (electronic supplementary material, table S3). In line with the sensitivity of this mutant, a hypoxia-repressed protein (AFUA_2G15290; ↓ −1.58155) with a glutathione-dependent formaldehyde-activating enzyme domain was significantly less abundant in this mutant. A predicted homocysteine *S*-methyltransferase (AFUA_3G01329) was uniquely detected in Δ*gliT*. This protein may be specifically upregulated in Δ*gliT* to counteract the homocysteine generated from BmGT generation.

**Table 2.** Top five proteins with increased abundance in *A. fumigatus* Δ*gliT*::Δ*gtmA* compared with *ΔgliT* following a 3 h exposure to gliotoxin (2.5 µg ml^−1^). Data sorted by fold change, in descending order.

protein description	log_2_(fold increase)	peptides	sequence coverage (%)	protein IDs
glutathione *S*-transferase encoded in the gliotoxin biosynthetic gene cluster, GliG	4.12873	10	61.2	AFUA_6G09690
N methyltransferase, encoded in the putative gliotoxin biosynthetic gene cluster, GliN	3.02483	19	89	AFUA_6G09720
conserved hypothetical protein, encoded in the putative gliotoxin biosynthetic gene cluster, GliH	2.86394	5	30.7	AFUA_6G09745
predicted *O*-methyltransferase, encoded in the putative gliotoxin biosynthetic gene cluster, GliM	2.78612	23	76.1	AFUA_6G09680
glutamyl-tRNA(Gln) amidotransferase, subunit A	2.16436	6	15.9	AFUB_092380
D-3-phosphoglycerate dehydrogenase, role in l-serine biosynthetic process	2.14692	13	34.2	AFUA_2G04490

### 2.7. Gliotoxin uptake is enhanced in Δ*gliT*::Δ*gtmA* compared with wild-type, Δ*gliT* or Δ*gtmA* following gliotoxin exposure

In the absence of GliT, *A. fumigatus* is unable to export gliotoxin and consequently unable to protect itself upon exposure. This suggests that GliA-mediated gliotoxin efflux is specific for the disulfide form of gliotoxin and that the dithiol form cannot be secreted. Based on this knowledge, we considered that Δ*gliT*::Δ*gtmA* may accumulate significantly more intracellular gliotoxin compared with Δ*gliT*. This would be due to the fact that GliA-mediated gliotoxin efflux is disabled in the absence of the gliotoxin oxidoreductase GliT [24]. The concomitant disruption of GtmA in this background may result in a combined inability to dissipate dithiol gliotoxin as BmGT, which would explain the high sensitivity of this double mutant to exogenous gliotoxin exposure. The detectable levels of extracellular gliotoxin and BmGT (GT/BmGT) in culture supernatants following 3 h gliotoxin exposure (2.5 µg ml^−1^) was measured across selected *A. fumigatus* mutants by RP-HPLC to observe if this correlated with the level of gliotoxin sensitivity (figure 5*b*). Notably, Δ*gliT*::Δ*gtmA* has significantly lower extracellular GT/BmGT than Δ*gliT* after 3 h (*p* = 0.0118). This strongly suggests that gliotoxin is accumulating intracellularly in this mutant at a higher level than Δ*gliT*, resulting in high sensitivity to this toxin.

### 2.8. Confocal microscopy reveals that GtmA-eGFP is localized throughout the cytosol following gliotoxin exposure

GliT::eGFP was previously shown to be localized in the cytoplasm and nuclei of *A. fumigatus*, supporting its role in protection against gliotoxin toxicity [4]. Construction of *A. fumigatus* GtmA::eGFP, which was also transformed with histone 2A monomeric red fluorescent protein fusion (H2A::mRFP) to visualize the nuclei, was undertaken (electronic supplementary material, figures S19 and S20). Confocal microscopy revealed that GtmA::eGFP accumulates in the cytosol of *A. fumigatus* following gliotoxin exposure (5 µg ml^−1^, 3 h; electronic supplementary material, figure S21). No eGFP signal was detectable in either the methanol control or the Δ*gtmA* strain exposed to gliotoxin. Cytosolic localization is a feature of methyltransferases involved in the detoxification of thiol compounds in other organisms. The thiol methyltransferase from *Euglena gracilis* [25] and the methyl chloride transferase from *E. muricata* [26] are also localized in the cytosol. The cytosolic location of these enzymes presumably allows for rapid substrate access. In contrast, the fungal methyl chloride transferase, which is not involved in detoxification, is membrane-bound [27].

## 3. Discussion

Herein, we reveal detailed structural, mechanistic and functional insights into GtmA-mediated gliotoxin *bis*-thiomethylation and reveal hitherto occluded roles of this enzymatic process, whereby it functions with GliT to dissipate dithiol gliotoxin in *A. fumigatus*. Structures of apo-GtmA, GtmA–SAH and GtmA–SAM in combination with activity assays provide new insights into this transformation. The deletion of *gtmA* in the *A. fumigatus* mutant backgrounds Δ*gliT* and Δ*gliA* uniquely reveals the consequence, and metabolic impact, of an uncontrolled dithiol gliotoxin biosynthetic capacity. For the first time, it is clear that both GtmA and GliT are necessary to prevent the deleterious effects of dithiol gliotoxin in an ETP producer.

Our data suggest that GtmA methylates dithiol gliotoxin by a non-processive mechanism. Examples of natural product methyltransferases that processively di- or trimethylate an acceptor substrate have recently been described. The ergothioneine biosynthetic methyltransferase EgtD catalyses three consecutive methyltransfers to the α-amino group of histidine. EgtD processivity is a result of increased affinity for the methylated intermediates, rather than increased catalytic efficiency of the second and third methylation steps [21]. Similarly, an iterative *O*-methyltransferase (FtpM) was recently shown to catalyse 1,11-dimethylation of *A. fumigatus* fumaric acid amides. No monomethylated derivatives were detectable in the conditions tested [28]. BamL, which dimethylates the N-terminus of PZN, does not release monomethylPZN during the reaction. However, following mutagenesis of Tyr182Phe in BamL, the enzyme primarily yields a monomethylated product instead of the dimethylated product [29].

The homodimeric *N,N*-dimethyltransferases Tylm1 and DesVI involved in the biosynthesis of the sugars mycaminose and desosamine appear to be the only other examples in the literature of non-processive, sequential natural product methyltransferases [30]. Chen *et al.* [30] state that the monomethylated intermediate is not an aberrant shunt product prematurely leaking from the enzyme active site, but a true intermediate during catalysis. Similar to our proposed reaction mechanism for GtmA, both the monomethylated intermediate and SAH are released from the active site of Tylm1 and DesVI following the first methylation reaction. Subsequent binding of another SAM cofactor and the monomethylated intermediate leads to the final product [31]. The proposed reaction mechanism is consistent with the finding that GtmA can bind SAM in the absence of its substrate dithiol gliotoxin. A key difference between GtmA and Tylm1 is that little monomethylated product accumulates in the Tylm1 reaction as the second methylation has a considerably greater reaction rate than the first [30]. In contrast, GtmA appears to preferentially methylate dithiol gliotoxin over the monomethylated intermediate (figure 3*a*,*b*).

We hypothesized that the lack of processivity may be rooted in the way GtmA deals with SAM/SAH. In order to investigate possible structural rearrangements and gain mechanistic insights into this transformation, we structurally elucidated the GtmA-apo, GtmA–SAM and GtmA–SAH-bound structures. These structures revealed several flexible parts of the upper domain (α1, α4 and β3) which are not visible in the electron density of the apo structure. This is probably a consequence of the dynamic nature of GtmA. The central region forms a helical-lid-like structure near the putative active site in GtmA [19]. We propose that this region controls the entry of SAM into, and the exit of SAH from, the active site of GtmA, and shapes the active site for substrate binding and catalysis. Indeed, these structural elements are stabilized in the GtmA–SAM and GtmA–SAH structures. The GtmA–SAH structure reveals a well-shaped hydrophobic cavity close to SAH, which probably represents the binding pocket. As described previously, residues Met10, Phe11, Ser131, His189, Tyr237 and Lys241 restrict access to the active site in this structure [19]. Based on molecular docking and an SAH-bound structure, Duell *et al*. [19] proposed that the second dithiol gliotoxin methylation occurs before the monoalkylated intermediate is released from the GtmA active site. However, a combination of our structural and RP-HPLC assay data strongly suggests that GtmA releases MmGT before proceeding to the second methylation step. This seems required, because access to the active site is restricted for both gliotoxin and SAH in the GtmA–SAH structure, suggesting that the binding pocket must be disrupted following the first methyltransfer to release MmGT. The recruitment of another SAM molecule then renders GtmA ready for the second round of methylation, leading to BmGT generation. Our GtmA–SAM structure provides evidence that SAM binding occurs in a structural intermediate state, where the putative gliotoxin and SAM binding pockets are open due to a dramatic movement of the lower domain and helix α1 (figure 2*a*,*b*). A characteristic of this intermediate is the finding that SAM binds in a solvent-exposed position, whereas SAH is almost completely buried in the SAH complex, which corroborates the hypothesis that the GtmA–SAM structure represents a conformation involved in SAM loading.

SAH has been shown to be a potent feedback inhibitor of SAM-dependent methyltransferases [32]. Interestingly, this inhibition does not occur for GtmA, which appears to be resistant to SAH-mediated inhibition. This is probably consequential to GtmA-mediated catalytic transfer of two methyl groups, which means that if SAH was capable of inhibiting the enzyme, this would affect its ability to rapidly bind a second SAM molecule for the second methyltransfer of MmGT. Similarly, the plantazolicin *N*-methyltransferase BamL, which dimethylates the N-terminus of this antibiotic, is also resistant to SAH by-product inhibition [33].

Disrupting the gliotoxin *bis*-thiomethylation ability of *A. fumigatus* did not increase the sensitivity of this organism to gliotoxin [12]. However, Δ*gliT*::Δ*gtmA* is more sensitive to exogenous gliotoxin in comparison with *A. fumigatus* Δ*gliT* or Δ*gtmA*. In addition, Δ*gliA*::Δ*gtmA* was shown to be significantly more sensitive to gliotoxin than Δ*gliA*. This implies that GliT activity effectively compensates for the absence of GtmA in *A. fumigatus* Δ*gtmA* and that GtmA may have been an ancestral protective strategy against gliotoxin and/or other ETPs prior to the acquisition of the gliotoxin cluster by this organism. The presence of MT-II in non-ETP producing *A. niger* supports this hypothesis as MT-II has been shown to be involved in protection against gliotoxin and possibly other ETPs [15]. Subsequent redundancy of this superseded protection mechanism in *A. fumigatus* may have resulted in the neofunctionalization of GtmA and its interlinked association with the gliotoxin biosynthetic gene cluster.

Δ*gliT* produces significantly more BmGT than wild-type or Δ*gliA* following exogenous gliotoxin exposure [24]. This suggested that the inability to oxidize dithiol gliotoxin to gliotoxin by GliT results in a higher substrate availability for GtmA. The severe SAM depletion noted in Δ*gliT* following gliotoxin exposure was alleviated in the double deletion mutant Δ*gliT*::Δ*gtmA*. *Aspergillus fumigatus* Δ*gliA*::Δ*gtmA* was also found to exhibit higher levels of cellular SAM than Δ*gliA* following gliotoxin exposure. Overall, these observations establish a direct link between the depletion of cellular SAM upon gliotoxin exposure and gliotoxin *bis*-thiomethylation mediated by GtmA. This result also highlights the complexity of the *A. fumigatus* response to this toxin. Considering the metabolic expense of *bis*-thiomethylation on SAM depletion in *A. fumigatus* Δ*gliT* and the fast, reversible nature of GliT-mediated dithiol gliotoxin oxidation, it is understandable that the process of dithiol *bis*-thiomethylation may have been relegated to a backup strategy in *A. fumigatus*.

Based on the significantly lower concentration of gliotoxin detected in the culture supernatants of Δ*gliT*::Δ*gtmA* exposed to gliotoxin, we hypothesize that following gliotoxin exposure, Δ*gliT*::Δ*gtmA* accumulates high levels of intracellular dithiol gliotoxin which cannot exit the cell by either oxidation (GliT) and subsequent efflux by GliA, or by GtmA-mediated *bis*-thiomethylation. This hypothesis is supported by the RP-HPLC-based gliotoxin uptake/efflux investigation. The gliotoxin sensitivity of *A. fumigatus* mutants is most significant where the removal of intracellular gliotoxin [12] is hampered by a combined deficit of the inability to oxidize, efflux or *bis*-thiomethylate this metabolite. Δ*gliT*::Δ*gtmA* was shown to have the lowest levels of extracellular gliotoxin present following 3 h gliotoxin exposure, which is in complete agreement with the inability of this mutant to directly dissipate any dithiol gliotoxin which enters the cell.

**Figure 6.**
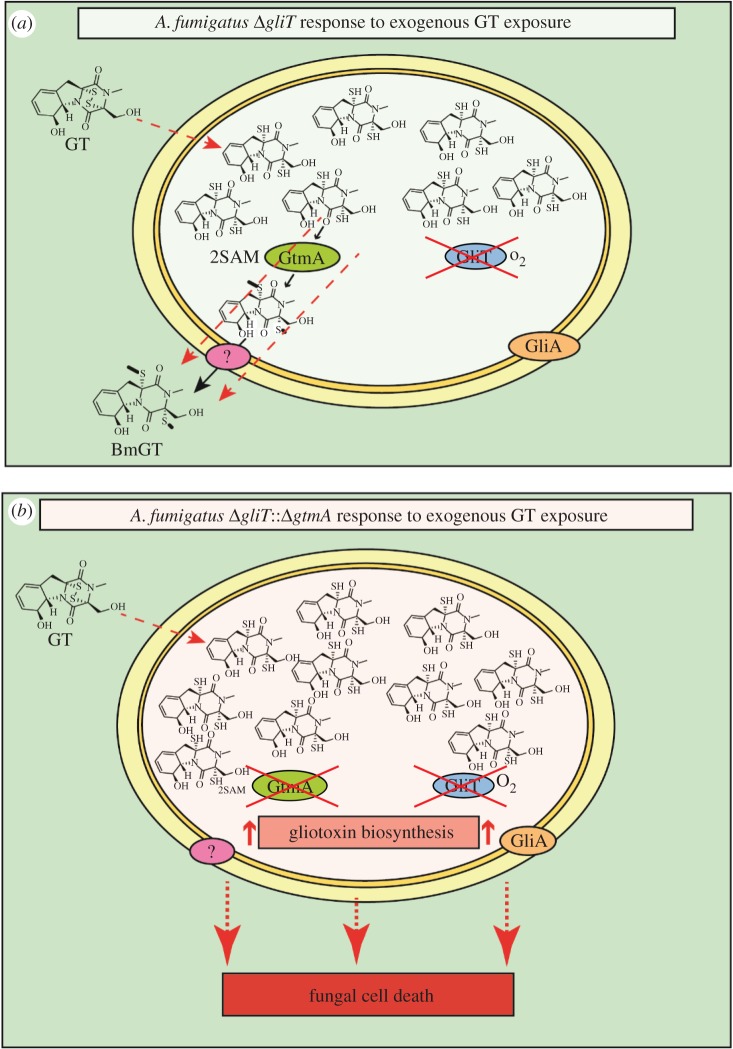
(*a*) *Aspergillus fumigatus* Δ*gliT* responds to exogenous gliotoxin by effecting excessive *bis*-thiomethylation on this metabolite, resulting in cellular SAM depletion. (*b*) The highly sensitive *A. fumigatus* Δg*liT*::Δ*gtmA* cannot remove intracellular accumulated dithiol gliotoxin by *S*-methylation [6,35] or oxidation. This results in cell death as a result of the combined effect of gliotoxin toxicity and *gli*-cluster over-activation owing to the sustained presence of dithiol gliotoxin.

Considering that gliotoxin exposure results in the induction of *gli*-cluster expression [34] (possibly by intracellular accumulation), the heightened sensitivity of Δ*gliT*::Δ*gtmA* may also be the result of the over-activation of the *gli*-cluster leading to a sustained abundance of cognate enzymes. Relevantly, upon exposure to gliotoxin, five gliotoxin biosynthetic enzymes (GliG, GliN, GliH, GliM and GliP) were found to be highly abundant in Δ*gliT*::Δ*gtmA* compared with Δ*gliT*. Thus, it is plausible that the gliotoxin pathway could proceed to the penultimate step (dithiol gliotoxin) before being trapped in the cell owing to the absence of both GliT and GtmA. It is likely that this *in vivo*-produced dithiol gliotoxin contributes to cellular oxidative stress in conjunction with the exogenously added gliotoxin, which, following uptake, undergoes intracellular GSH-mediated reduction, hampering its export from the cell (figure 6).

Gliotoxin may act as a specific elicitor of secondary metabolism as shown in other systems, but the chemical and molecular complexity of these induction processes means that the precise mechanism of activation is poorly understood. Sixteen of the 95 proteins significantly altered in abundance in Δ*gliT*::Δ*gtmA* exposed to gliotoxin are uncharacterized, five of which (AFUA_1G11120, AFUA_1G11780, AFUA_1G14500, AFUA_1G15260 and AFUA_5G06370) have no conserved domains or motif hits. AfuVipB, a putative H3-K9-specific histone methyltransferase, was significantly downregulated in Δ*gliT*::Δ*gtmA* exposed to gliotoxin (AFUA_3G14920; ↓ −1.4122). Induction of the orsellinic acid gene cluster in *A. nidulans* by the bacterium *Streptomyces rapamycinicus* was shown to be dependent on the Saga/Ada complex containing the histone acetyltransferase proteins GcnE and AdaB. A Saga/Ada-dependent increase of histone 3 acetylation at lysine 9 and 14 was shown to occur during this interaction between the fungus and bacterium. However, the exact nature of the bacterial signal remains to be elucidated [36]. It is conceivable that gliotoxin-mediated induction of the *A. fumigatus gli*-cluster may occur through a related mechanism.

Considering the ability of ETP toxins to rapidly generate deleterious reactive oxygen species within the cell it may be an evolutionary adaptation of ETP exposed organisms to disable ETPs with a rapid, single *S*-methylation instead of holding the substrate for *bis*-thiomethylation. Processively modifying both thiols on the same ETP molecule could be seen as a disadvantage in this scenario. In fact, epimonothiodiketopiperazine derivatives have been reported to be at least one order of magnitude less active than their disulfide forms [7]. This reaction mechanism may also facilitate a more generalized defence against a variety of thiol-containing natural products.

Detailed analysis of GtmA, the SAM-dependent gliotoxin *bis*-thiomethyltransferase that negatively regulates gliotoxin biosynthesis, reveals extensive structural rearrangement upon cofactor binding, which provides new insights into the functionality of this enzyme class. Complete disruption of self-protection and negative regulatory systems, respectively, significantly augment hypersensitivity to gliotoxin in *A. fumigatus* owing to dysregulated *gli*-cluster activity, dithiol gliotoxin biosynthesis and extensive compensatory proteomic remodelling. We conclude that *bis*-thiomethylation, an ancestral protection system against redox-active metabolites, has undergone functional evolution.

## 4. Material and methods

### 4.1. Protein expression

Expression plasmids pEX-N-GST-GtmA (OriGene) and pET19 m-GtmA (modified pET19b, Novagen) were freshly transformed into chemically competent *Escherichia coli* BL21 (DE3) cells and subsequently used for pre-culture inoculation in lysogenic broth (LB) supplemented with 100 mg l^−1^ ampicillin. Large-scale production of the native protein was carried out in terrific broth including the required antibiotics. Cells were incubated at 37°C and 120 rpm until optical density reached 0.6–0.8 followed by a temperature reduction to 20°C. The induction of heterologous gene expression was performed by incubating with 500 µM IPTG for another 20 h. Cells were collected by centrifugation at 5000 *g* for 10 min.

l-seleno-methionine-labelled GtmA was produced by using minimal medium (M9) as described elsewhere [37]. The LB pre-culture containing pEX-N-GST-GtmA *E. coli* BL21 (DE3) was centrifuged and washed twice with M9 minimal medium before inoculation of a larger culture in M9 medium with corresponding antibiotics at 37°C and 120 rpm. An amino acid cocktail comprising 100 mg l^−1^ lysine, 100 mg l^−1^ phenylalanine, 100 mg l^−1^ threonine, 50 mg l^−1^ isoleucine, 50 mg l^−1^ leucine and 50 mg l^−1^ valine was added to the cell suspension to suppress methionine biosynthesis when the optical density at 600 nm reached 0.5. After an additional 15 min incubation 60 mg l^−1^ l-seleno-methionine and 500 µM IPTG were added to the cells and the culture was further incubated at 20°C for 20 h.

### 4.2. Protein purification

The cells were diluted with lysis buffer (50 mM Na_2_HPO_4_ pH 8.0; 300 mM NaCl; 5 mM imidazole, 1 mM PMSF, 1 µg ml^−1^ pepstatin A and 300 µM lysozyme), subsequently lysed using an EmulsiFlex-C3 homogenizer (AVESTIN) and centrifuged for 30 min at 30 000*g* and 4°C. The resulting supernatant was applied onto a HisTrap chelating column (GE Healthcare Life Science) loaded with 100 mM nickel sulfate equilibrated in buffer A (50 mM Na_2_HPO_4_ pH 8.0, 300 mM NaCl, 5 mM imidazole). Contaminating proteins were washed from the column with buffer A until absorption at 280 nm reached the baseline. Elution of GtmA was performed by using a linear gradient over 20 column volumes (CV) to a final concentration of 100% buffer B (buffer A with 200 mM imidazole). To remove the affinity tag, TEV-protease cleavage was carried out with a 1 : 40 molar ratio (protease to protein) overnight at 4°C during dialysis against buffer C (50 mM Tris–HCl pH 7.4; 50 mM NaCl). The cleaved GtmA was separated from the protease and the affinity tag with a second nickel-loaded HisTrap chelating column (GE Healthcare Life Science) using buffer C and buffer D (buffer C with 200 mM imidazole). The flow through was collected, concentrated and applied to an S200 26/60 size exclusion column (GE Healthcare Life Science) equilibrated with buffer C. Pure protein fractions were collected, concentrated to 20 mg ml^−1^ and flash frozen until needed for further experiments.

The l-seleno-methionine-labelled GtmA was purified as an N-terminally GST-tagged fusion protein as described elsewhere [12]. An additional ion exchange chromatography step using a Q-Sepharose column (GE Healthcare Life Science) equilibrated in buffer ‘low salt’ (20 mM Tris–HCl pH 8.5, 20 mM NaCl) was used to separate the TEV protease and residual GST from GtmA. Elution was carried out with a linear gradient over 32 CV and a final concentration of 1 M NaCl using buffer ‘high salt’ (buffer ‘low salt’ with 1 M NaCl). Fractions containing l-seleno-methionine-labelled GtmA were collected and applied to an S200 26/60 size exclusion column equilibrated with buffer C as a final purification step.

### 4.3. Site-directed mutagenesis of gtmA

The pET19m_GtmA vector served as a template for *in vitro* site-directed mutagenesis using the QuikChange XL Site-Directed Mutagenesis Kit (Stratagene) and the oligonucleotides used are listed in electronic supplementary material, table S4. Mutagenesis was carried out as stated in the supplied protocol. Hereby, the plasmids pET19m_GtmA_W157V, pET19m_GtmA_W162V, pET19m_GtmA_N159V, pET19m_GtmA_F185G and pET19m_GtmA_F127V were created. The resulting plasmids were propagated in *E. coli* DH5α cells. The DNA sequence was verified by sequencing, and the vectors were introduced into *E. coli* BL21 (DE3) cells for protein overproduction.

### 4.4. Methyltransferase assay of GtmA mutants using RP-HPLC

GtmA methyltransferase activity of the wild-type GtmA and mutants was monitored by RP-HPLC in 50 mM Tris–HCl, 50 mM NaCl, pH 7.4 using 750 µM SAM and 300 µM dithiol gliotoxin. 1 µM of purified enzyme was added to each reaction. The samples were incubated at 37°C for 1 h prior to RP-HPLC analysis. For GtmA activity analysis under SAM limiting conditions, a range of SAM concentrations between 100 and 400 µM were used. For these experiments, reaction mixtures were directly injected onto the RP-HPLC column without quenching in order to ensure that enzyme-bound gliotoxin-ligands were not released. For *K*_m_ determination, substrates (either dithiol gliotoxin or MmGT) from 31.25–750 µM were separately combined with SAM (1.5 mM) and mixed at 37°C, in duplicate. GtmA (10 µM final) was added and mixed rapidly, and reactions were stopped after 15 s by addition of TCA to 15% (w/v). Samples were incubated on ice for 20 min, centrifuged to remove precipitated protein and analysed by RP-HPLC with detection at 254 nm. *K*_m_ and nonlinear curve determination was done using GraphPad Prism.

### 4.5. Crystallization, data collection and refinement

Crystal screening was carried out with an automated crystallization robot (Zinsser Analytic) by using the sitting drop vapour diffusion method and mixing 0.2 µl protein and 0.2 µl reservoir solution at 20°C and 4°C. Well diffracting protein crystals of apo-GtmA were obtained by mixing 15 mg ml^−1^ protein with reservoir containing 0.2 M sodium formate and 20% (w/v) PEG 3350 at 4°C, whereas the l-seleno-methionine-labelled protein crystallized in reservoir comprising 0.2 M calcium acetate hydrate and 20% (w/v) PEG 3350 at a concentration of 15 mg ml^−1^ and 20°C. Crystals of the SAH and SAM complex were achieved by incubation of 15 mg ml^−1^ GtmA with 0.5 mM SAH and 12.5 mM SAM for 30 min before mixing with 2.4 M (NH_4_)_2_SO_4_, 0.1 M MES pH 6.0 and 0.2 M sodium chloride, 0.1 M sodium phosphate citrate pH 4.2, 22.3% (w/v) PEG 8000, 0.08% (w/v) ellipticine, 0.20% (w/v) gibberellin A_3_, 0.20% (w/v) trans-cinnamic acid, 0.20% (w/v) phenol, 0.20% (w/v) succinic acid disodium salt hexahydrate and 0.02 M HEPES pH 6.8, respectively. The SAM-GtmA crystal was further soaked with 100 mM Yb-DO3A (Lanthanide Phasing Kit, Jena Bioscience) for 10 s. All crystals were cryo-protected by using 10% (w/v) glycerol. Diffraction data of the l-seleno-methionine-labelled GtmA were collected on beamline BL14.2 operated by the Helmholtz-Zentrum Berlin at the BESSY II electron storage ring (Berlin-Adlershof, Germany) [38], of the apo GtmA and the SAH complex on beamline ID 30B at European Synchrotron Radiation Facility (Grenoble, France) [39] and of the SAM complex on beamline P11 at DESY, PetraIII (Hamburg, Germany) [40]. Single anomalous diffraction data of the l-seleno-methionine-labelled GtmA and of the Yb-soaked SAM complex were collected at a wavelength of 0.9798 Å and 1.3855 Å, whereas native data of apo GtmA and the SAH complex were collected at 0.9762 Å. All diffraction data were indexed and integrated with XDS [41] and scaled with AIMLESS from the CCP4 package [42]. For the Se-SAD data, the AUTOSOL [43,44] routine of the PHENIX software suite [43] was used to generate experimental phases and to build the structure. The resulting model was used for rigid body refinement in phenix.refine to solve the structure of native GtmA. The SAM complex structure was solved with MR-Yb-SAD by using the apo structure as model in PHASER [43,45], built with AUTOBUILD [43,46] and finally used to solve the SAH complex with PHASER [45]. All structures have been refined using alternating steps of manual adjustment in Coot [47] and maximum-likelihood refinement in PHENIX [43], and finally validated by using MolProbity [48].

Coordinates and diffraction data have been deposited in the Protein Data Bank [49] with accession codes 5JGJ, 5JGK and 5JGL for the GtmA-apo, SAH and SAM complexes, respectively.

### 4.6. Microscale thermophoresis

To determine the binding affinity between GtmA and SAM or SAH, MST was used [50]. For this, GtmA was labelled with Cy5 Mono NHS Ester (Amersham) according to the vendor's manual. To determine *K*_D_-values for SAM and SAH, a fixed concentration of 100 nM GtmA-Cy5 was titrated with serial 1 : 1 dilutions of both ligands in labelling buffer (50 mM Tris, 50 mM NaCl, pH 7.4, 0.5 mg ml^−1^ bovine serum albumin). In order to determine *K*_D_-values for GtmA and SAM under oxidizing and reducing conditions, 10 mM oxidized glutathione (GSSG; oxidizing conditions) or 10 mM TCEP (Tris(2-carboxyethyl)phosphine, reducing conditions) was added to the labelling buffer. The experiments were performed using a NanoTemper Monolith NT.115 instrument with standard (for non-binding molecules) capillaries at 22°C, 20% LED power and 40% MST power. For *K*_D_ determination, experiments were executed in triplicate. The response value was averaged and plotted against the concentration of the ligands. *K*_D_ values were extracted by fitting to the quadratic equation (4.1), using the vendor's software (*K*_D_ fitted in MO.Affinity Analysis software, NanoTemper).4.1
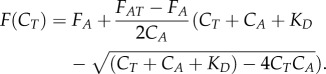


Here, *F_A_* represents the response value of unbound labelled molecules, *F_AT_* the response value of the complex of labelled and the unlabelled ligand molecules, *C_A_* the concentration of labelled molecule and *C_T_* the concentration of the unlabelled ligand molecule.

### 4.7. Generation of *Aspergillus fumigatus* Δ*gliT::*Δ*gtmA* and Δ*gliA::*Δ*gtmA*

*Aspergillus fumigatus gtmA* double mutants were generated in the Δ*gliT* and Δ*gliA* backgrounds via the bipartite marker technique using the hygromycin (*hph*) resistance marker for deletion (electronic supplementary material, experimental procedures). Primers used for generating all deletion constructs are given in electronic supplementary material, table S4.

### 4.8. RP-HPLC and LC–MS detection of natural products from *Aspergillus fumigatus* culture supernatants

For gliotoxin spiking experiments, cultures were grown in Sabouraud–dextrose medium for 21 h followed by gliotoxin addition (2.5 µg ml^−1^ final) for 3 h (*n* = 3 biological replicates for all specimens). All strains used are given in electronic supplementary material, table S5. Organic extracts from supernatants were analysed by RP-HPLC with UV detection (Agilent 1200 system), using a C18 RP-HPLC column (Agilent Zorbax Eclipse XDB-C18 Semi-Preparative; 5 µm particle size; 4.6 × 250 mm) at a flow rate of 2 ml min^−1^. A mobile phase of water and acetonitrile with TFA was used under various gradient conditions. *Aspergillus fumigatus* wild-type, deletion and complementation strains were grown for 72 h in Czapek Dox medium (unless stated otherwise) followed by organic extraction and LC–MS analysis as previously described [12]. Supernatants were diluted 1/10 in 0.1% (v/v) formic acid and spin filtered prior to LC–MS analysis (Agilent Ion Trap 6340) to detect BmGT presence. Gliotoxin (purity: 98%) and BmGT (purity: 99%) standards were obtained from Sigma-Aldrich and Enzo Life Sciences, respectively.

### 4.9. *Aspergillus fumigatus* phenotypic assays

*Aspergillus fumigatus* wild-type and mutant strains were grown on MEA agar for 5 days at 37°C after which conidia were harvested. Conidia were serially diluted to 10^−2^ and 10^−4^ in PBS. Aliquots (5 µl) of each dilution were spotted onto agar plates containing gliotoxin. Plates were incubated at 37°C and growth was monitored at specific time intervals by measuring the diameter of radial growth (cm) of each colony. Two-way ANOVA analysis was performed to determine the statistical significance between strains on the various additives.

### 4.10. Detection and quantification of *S*-adenosylmethionine

Czapek Dox medium was inoculated with 10^6^ ml^−1^ conidia (from *A. fumigatus* wild-type, gene deletion and complementation strains), in triplicate, and incubated at 37°C, shaking 200 rpm, for 21 h. Gliotoxin (5 µg ml^−1^ final) or methanol control was added and the cultures were incubated for a further 3 h before mycelia were harvested and snap frozen in liquid N_2_. SAM was extracted using a modified protocol as described previously [24]. Briefly, mycelia were ground under liquid N_2_ using a pestle and mortar. 0.1 M HCl (250 µl) was added to mycelia (100 mg) and incubated on ice for 1 h with regular vortexing. Following centrifugation at 13 000*g*, protein was removed from the supernatant by TCA precipitation. Samples were diluted in 0.1% (v/v) formic acid and analysed by LC–MS/MS using a porous-graphitized carbon chip on an Agilent 6340 ion-trap LC mass spectrometer (Agilent Technologies) using electrospray ionization.

### 4.11. Comparative quantitative proteomic analysis of *Aspergillus fumigatus* wild-type and mutant strains

*Aspergillus fumigatus* wild-type (ATCC26933), Δ*gtmA* and Δ*gliT* strains were cultured in Sabouraud–dextrose medium for 21 h followed by gliotoxin (2.5 µg ml^−1^ final) or methanol addition for 3 h (*n* = 3 biological replicates for all specimens). Mycelia were then harvested and snap frozen in liquid N_2_.

Mycelial lysates were prepared in lysis buffer (100 mM Tris–HCl, 50 mM NaCl, 20 mM EDTA, 10% (v/v) glycerol, 1 mM PMSF, 1 µg ml^−1^ pepstatin A, pH 7.5) with grinding, sonication and clarified using centrifugation. The resultant protein lysates were precipitated using trichloroacetic acid/acetone and resuspended in 100 mM Tris–HCl, 6 M urea, 2 M thiourea, pH 8.0. After dithiothreitol reduction and iodoacetamide-mediated alkylation, sequencing grade trypsin combined with ProteaseMax surfactant was added [51]. All peptide mixtures were analysed via a Thermo Fisher Q-Exactive mass spectrometer coupled to a Dionex RSLCnano. LC gradients ran from 4% to 35% B over 2 h, and data were collected using a Top15 method for MS/MS scans. Comparative proteome abundance and data analysis was performed using MaxQuant software (v. 1.3.0.5) [52], with Andromeda used for database searching and Perseus used to organize the data (v. 1.4.1.3) [12].

### 4.12. Analysis of *Aspergillus fumigatus* Δ*gtmA*::*gtmA-eGFP::H2A::mRFP* ^26933^ by confocal microscopy

The *gtmA*-eGFP strain transformed with H2A::mRFP (1 × 10^4^ conidia) was inoculated into 400 µl Czapek Dox medium in a Lab-Tek chambered borosilicate coverglass system. The chamber was incubated at 37°C for 21 h and then gliotoxin (5 µg ml^−1^ final) was added to the chambers. An equivalent volume of methanol was added to the control wells. As a second control a well containing Δ*gtmA* with gliotoxin was also prepared. Samples were incubated at 37°C for 3 h. Samples were viewed with an Olympus Fluoview 1000 confocal microscope.

## Supplementary Material

Supplementary Information

## Supplementary Material

Supplementary Data Table S3
