# Supplementary material for: Structural, mechanistic and functional insight into gliotoxin bis-thiomethylation in Aspergillus fumigatus
Source: Open Biol. 2017 Feb 8;7(2):160292. doi: 10.1098/rsob.160292 (PMC5356443; doi:10.1098/rsob.160292)
Supplement: Supplementary Information [file rsob160292supp1.docx]

**Running head:** GtmA - Structure and function.

**Structural, mechanistic and functional insight into gliotoxin *bis*-thiomethylation in *Aspergillus fumigatus*.**

Stephen K. Dolan^1,2^, Tobias Bock^3^, Vanessa Hering^3^, Rebecca A. Owens^1^, Gary W. Jones^1^, Wulf Blankenfeldt*^,3,4^ and Sean Doyle*^,1^.

^1^Department of Biology, Maynooth University, Maynooth, Co. Kildare, Ireland

^2^Current Affiliation: Department of Biochemistry, University of Cambridge, Cambridge CB2 1QW, UK

^3^Helmholtz Centre for Infection Research, Structure and Function of Proteins, Inhoffenstraße 7, 38124 Braunschweig, Germany.

^4^Institute of Biochemistry, Biotechnology and Bioinformatics, Technische Universität Braunschweig, Spielmannstr. 7, 38106 Braunschweig, Germany.

***Corresponding authors**

Professor Sean Doyle, Department of Biology, Maynooth University, Maynooth, Co. Kildare, Ireland.

Tel: +353-1-7083858; Fax: +353-1-7083845; E-mail: [sean.doyle@nuim.ie](mailto:sean.doyle@nuim.ie); Web: <http://biology.nuim.ie>

Professor Wulf Blankenfeldt, Structure and Function of Proteins, Helmholtz Centre for Infection Research, Inhoffenstraße 7, 38124 Braunschweig, Germany.

Tel: +49-531-61817000; Fax: +49-531-61817099; E-mail: [wulf.blankenfeldt@helmholtz-hzi.de](mailto:wulf.blankenfeldt@helmholtz-hzi.de) ; Web: <http://www.helmholtz-hzi.de/sfpr>

**Table S1.** Data collection statistics for GtmA and complexes.

Values in parentheses are for the highest resolution shell. All data sets were collected from single crystals

| Dataset | GtmA apo | SeMet-GtmA | complex with **SAM** | complex with **SAH** |
| --- | --- | --- | --- | --- |
| Wavelength (Å) / beamline^≠^ | 0.9762 / ESRF, ID30B | 0.9798 / BESSY, BL14.2 | 1.3855 / PETRAIII, P11 | 0.9762 / ESRF, ID30B |
| Resolution range (Å) | 47– 1.66 (1.69 – 1.66) | 47 – 2.20 (2.27 – 2.20) | 44 – 2.28 (2.37 – 2.28) | 45 -1.33 (1.35 – 1.33) |
| Space group | P6_2_ | P6_2_ | P12_1_1 | P12_1_1 |
| Unit cell parameters (Å) | 94.7, 94.7, 53.7 | 94.7, 94.7, 53.7 | 46.1, 114.1, 56.5 104° | 48.7, 109.3, 59.9 112° |
| Mosaicity (°)^†^ | 0.07 | 0.17 | 0.13 | 0.08 |
| Total No. of measured reflections | 629629 (29881) | 476759 (41831) | 501928 (40328) | 852355 (42805) |
| Unique reflections | 32033 (1598) | 14134 (1222) | 25675 (2614) | 130776 (6476) |
| Multiplicity | 19.7 (18.7) | 33.7 (34.3) | 19.5 (15.4) | 6.5 (6.6) |
| Mean I/σ(I) | 29.7 (2.3) | 42.5 (5.7) | 20.5 (4.5) | 14.4 (2.4) |
| Completeness (%) | 100 (100) | 100 (100) | 99.6 (96.8) | 98.7 (99.1) |
| R_meas_ (%)^‡^ | 5.2 (150) | 7.8 (89.2) | 29.4 (242) | 7.1 (97.4) |
| R_pim_ (%)^$^ | 1.5 (48.5) | 1.4 (21.3) | 6.5 (59) | 3.8 (52.7) |

^≠^ESRF: European Synchrotron Radiation Facility (Grenoble, France), BESSY: Berlin Electron Storage Ring for Synchrotron Radiation (Helmholtz Zentrum Berlin, Germany), PETRA III: Position-electron tandem-ring facility (Hamburg, Germany)

^†^Mosaicity values reported by AIMLESS [1]

^+^Values in parentheses refer to the highest resolution shell.

^‡^R_meas_ = Σ_hkl_ (N/(N – 1))^1/2^ Σ_i_ | I_i_ (hkl) – < I (hkl) > | / Σ_hkl_ Σ_i_ I_i_ (hkl), where N is the number of observations of the reflection with index hkl and I_i_ is the intensity of its i^th^ observation.

^$^R_pim_ = Σ_hkl_ (1/(N – 1))^1/2^ Σ_i_ | I_i_ (hkl) – < I (hkl) > | / Σ_hkl_ Σ_i_ I_i_ (hkl).[2]

**Table S2.** Refinement statistics for GtmA and complexes.

Values in parentheses are for the highest resolution shell.

| Dataset | GtmA apo | complex with **SAM** | complex with **SAH** |
| --- | --- | --- | --- |
| Resolution range (Å) | 44 – 1.66 (1.72 – 1.66) | 42 – 2.28 (2.36 – 2.28) | 45 – 1.33 (1.38 – 1.33) |
| R_cryst_ (%) | 16.8 (24.5) | 19.2 (28.4) | 13.9 (18.8) |
| R_free_ (%) | 18.6 (27.9) | 23.4 (30.8) | 16.6 (24.5) |
| No. of non-H atoms  Protein  Ion  Ligand  Water | 1812  -  -  131 | 4163  2  54  52 | 4448  25  52  401 |
| R.m.s. deviations  Bonds (Å)  Angles (°) | 0.017  1.352 | 0.003  0.621 | 0.007  1.024 |
| Average B factors (Å^2^)  Protein  Ion  Ligand  Water | 55  -  -  47 | 39  29  32  38 | 20  46  15  30 |
| Ramachandran plot  Favoured regions (%)  Outliers (%) | 97.4  0.00 | 96.8  0.00 | 99.1  0 |
| MolProbity score^#^ | 1.44 | 1.14 | 1.00 |
| PDB entry code | 5JGJ | 5JGL | 5JGK |

^#^As reported by MolProbity at <http://molprobity.biochem.duke.edu/> [3].

**Table S4.** Oligonucleotide primers used in the study.

| **Primer Name** | **Sequence (5’ to 3’)** |
| --- | --- |
| Δ*gtmAHYG*-P1 | ACCCTCATAGCTGCGACTTC |
| Δ*gtmAHYG*-P2 | GACTCGAGGGTCAACAAAGCTCTTGGTC |
| Δ*gtmAHYG*-P3 | GACCTGCAGGAAGACACCTATGGGGCGAAT |
| Δ*gtmAHYG*-P4 | GCAAAGCACTGAACAGCAAC |
| Δ*gtmAHYG*-P5 | CGGTTTGCGACTAAGCAGTT |
| Δ*gtmAHYG*-P6 | ACAGTGACAGTCCCGACGTT |
| HYoptrA1 | GCTCCATACAAGCCAACCAC |
| YGoptrA2 | GTCCTGCGGGTAAATAGCTG |
| *gtmA*_*eGFP*_F | TAGGTACCCGGTTTGCGACTAAGCAGT |
| *gtmA*_*eGFP*_R | TACCCGGGTCTGGGCTTGAGGCCGGTT |
| rGtmA-*HindIII* | GCAAGCTTATGTCCAAGTCAGACTACATCCA |
| rGtmA-*XhoI* | ATCTCGAGCTAGGGCTTGAGGCCG |
| GtmA_W157V_F | GGAATCCAGTTGAAGTTCTGCACTGTTGAGCTTGCAAGTATAC |
| GtmA_W157V_R | GTATACTTGCAAGCTCAACAGTGCAGAACTTCAACTGGATTCC |
| GtmA_W162V_F | GCGGCTTTCATAATTGGAATCACGTTGAAGTTCTGCCATGTTG |
| GtmA_W162V_R | CAACATGGCAGAACTTCAACGTGATTCCAATTATGAAAGCCGC |
| GtmA_N159V_F | CATAATTGGAATCCAGTTGAAGACCTGCCATGTTGAGCTTGCAAG |
| GtmA_N159V_R | CTTGCAAGCTCAACATGGCAGGTCTTCAACTGGATTCCAATTATG |
| GtmA_F185G_F | CGGCGTTGTGAAGAGCGATGCCCTCTTTCTGTGTTGGAAAC |
| GtmA_F159G_R | GTTTCCAACACAGAAAGAGGGCATCGCTCTTCACAACGCCG |
| GtmA_F127V_F | AAGCTCTGGAAGCCACCAGCGACAAAAACGTGCGTATAATG |
| GtmA_F127V_R | CATTATACGCACGTTTTTGTCGCTGGTGGCTTCCAGAGCTT |

**Table S5.** *A.fumigatus* strains used in the study.

**Strain Reference**

*A. fumigatus* ATCC26933 <http://www.atcc.org/>

*A. fumigatus*∆*gliT*^26933^ Schrettl et al. [4]

*A. fumigatus*∆*gtmA*^26933^ Dolan et al. [5]

*A. fumigatus* ∆*gliA*^26933^  Owens et al. [6]

*A. fumigatus* ∆*gtmA:gtmA* (*gtmA*^c^) Dolan et al. [5]

*A. fumigatus* Δ*gliT::*Δ*gtmA*^26933^ This work

*A. fumigatus* Δ*gliA::*Δ*gtmA*^26933^ This work

*A. fumigatus* Δ*gtmA*::*gtmA-eGFP::H2A::mRFP*^26933^ This work

**A.**


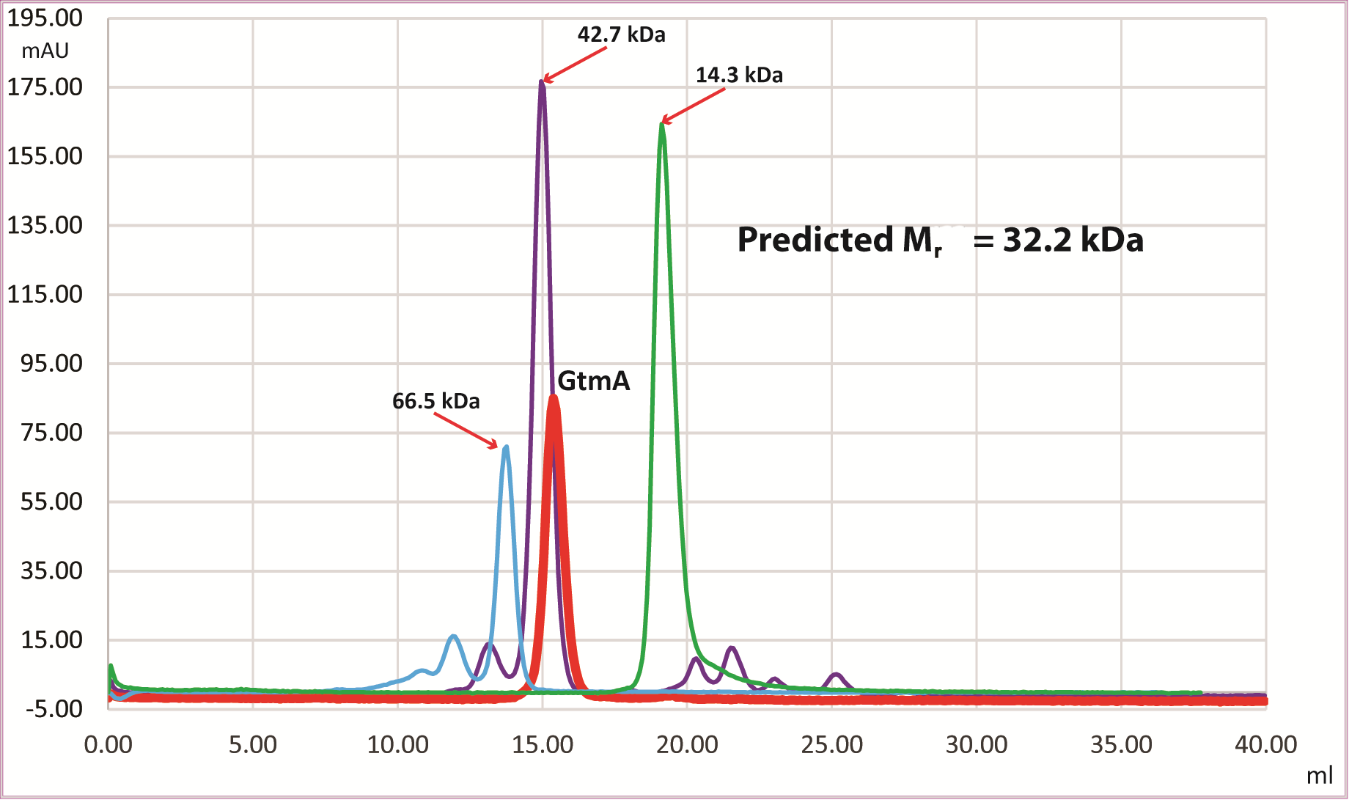


**B.**


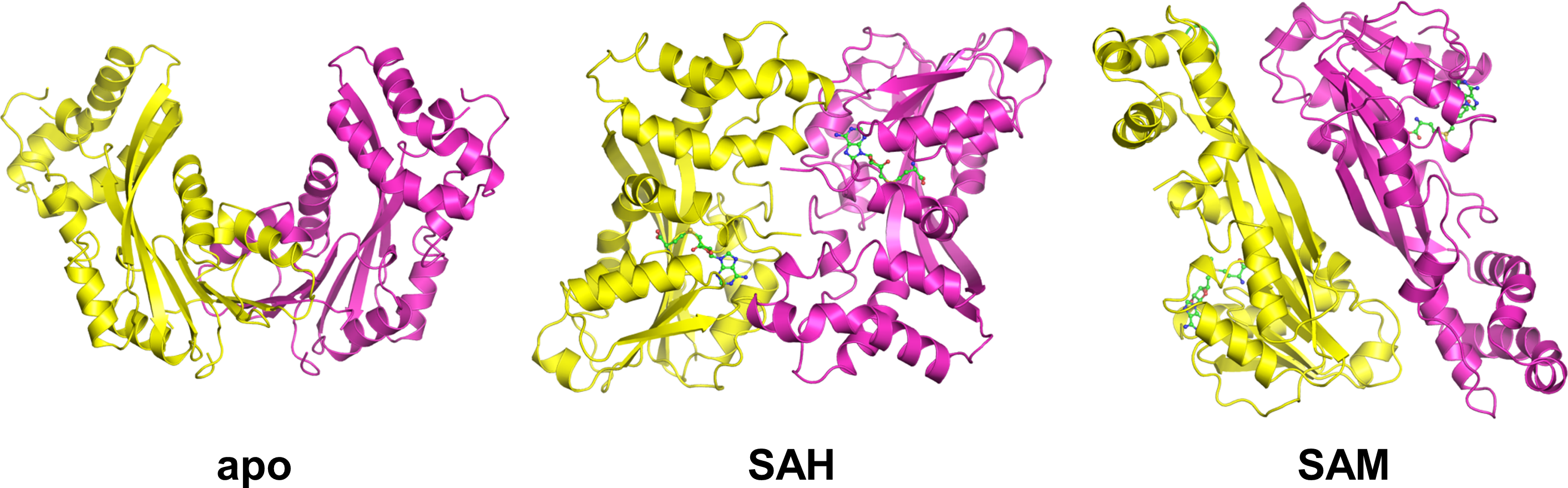


**Figure S1.** **A.** Size exclusion chromatography analysis of GtmA alongside commercial protein standards (GE Healthcare Life Sciences) revealed that GtmA protein is present as a monomer. Purified GtmA was loaded onto a Superdex 200 10/300 GL column (GE Healthcare, Germany) and appeared as a single, monodisperse peak. A selection of molecular mass calibrants (BSA, Ovalbumin and Lysozyme) was also individually applied to the column in order to uncover the oligomeric state of the methyltransferase. GtmA eluted after the BSA (66.5 kDa) and Ovalbumin (42.7 kDa) proteins, indicating that GtmA is present in monomeric form in solution. **B.** Dimeric arrangements of GtmA in the three crystal forms. See Table S1.


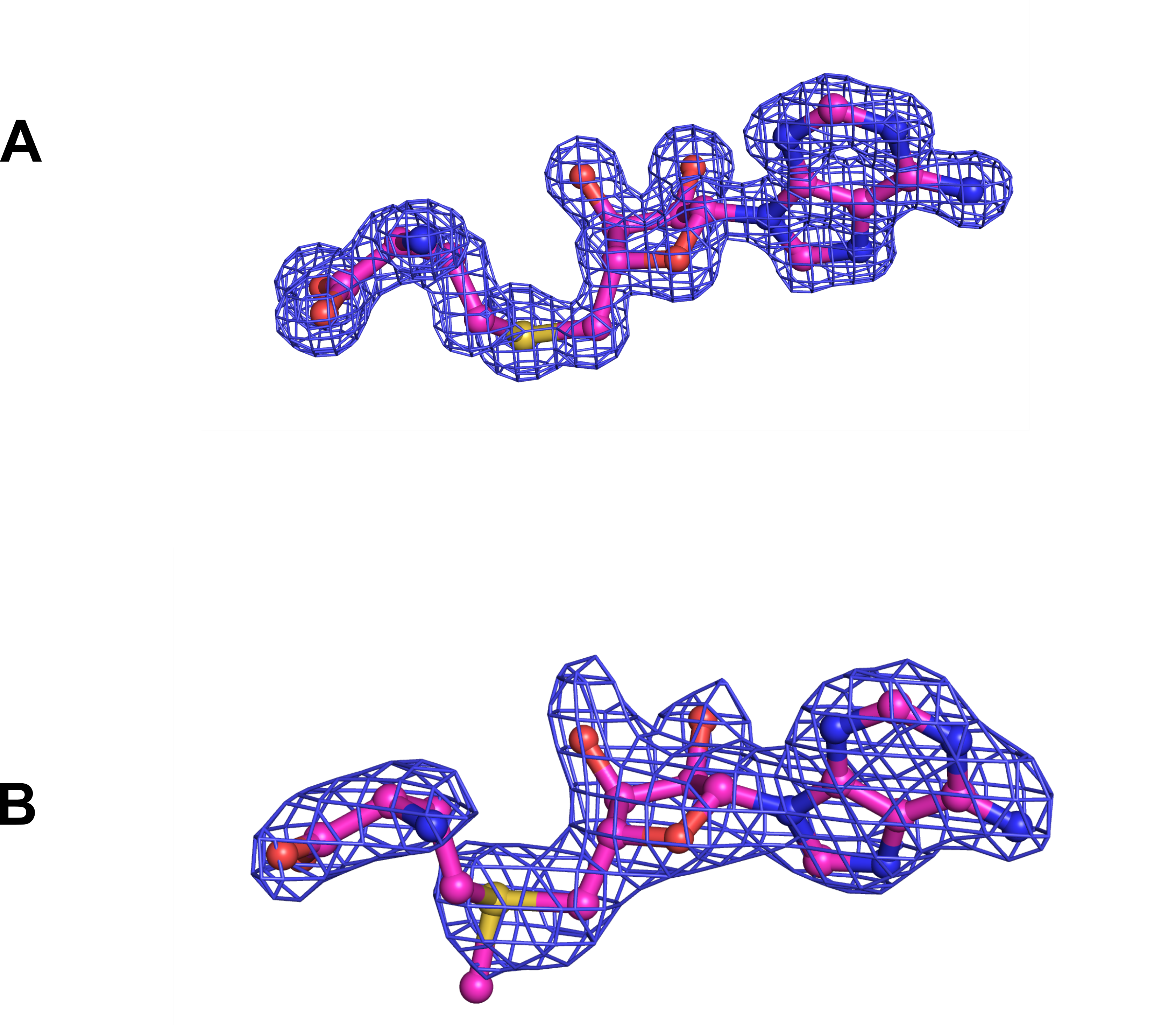


**Figure S2.** Difference electron density of SAM and SAH before placing the ligand. The difference electron density of **A.** SAH and **B.** SAM contoured at 3 σ.


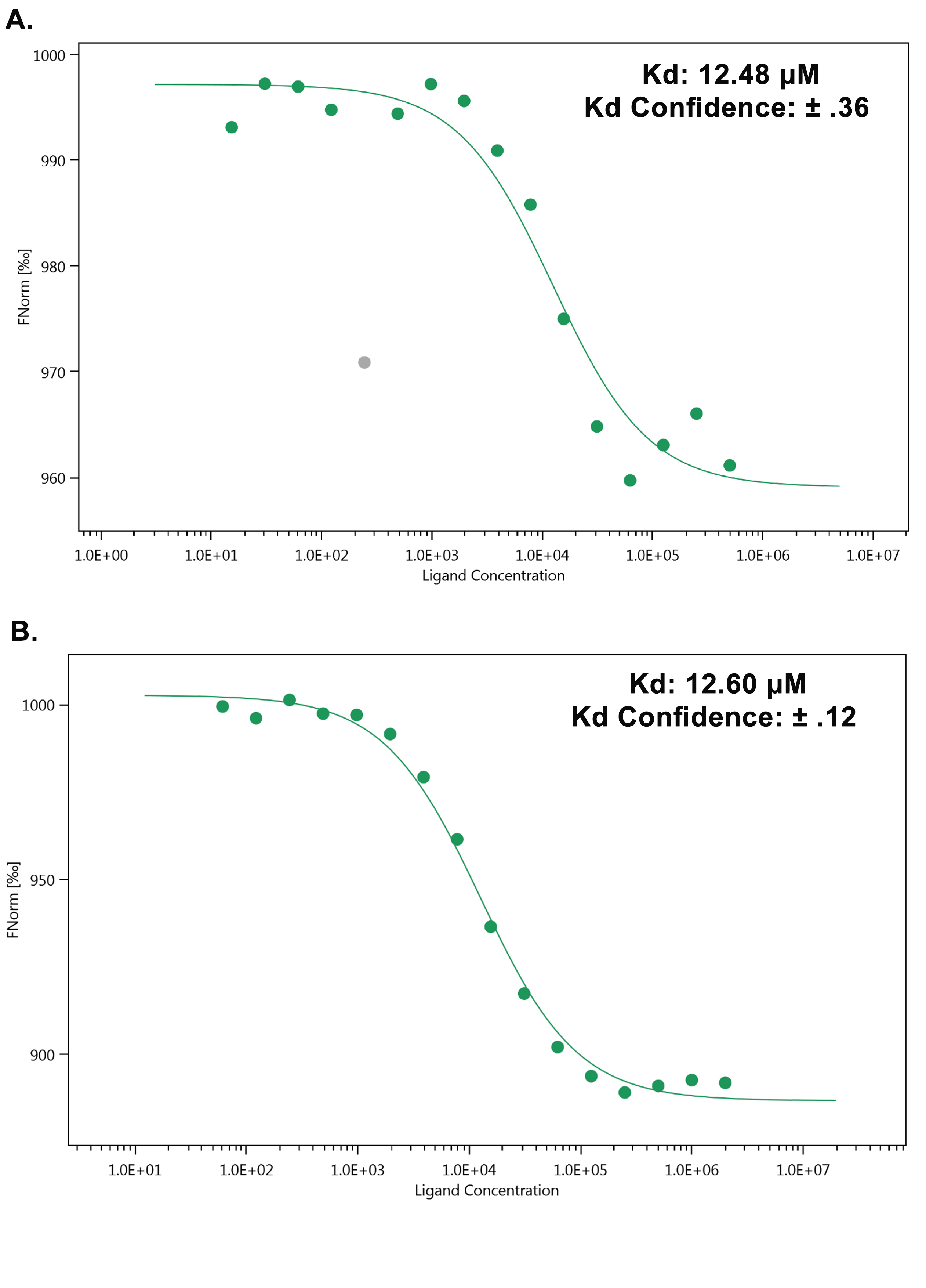


**Figure S3.** Microscale thermophoresis (MST) of GtmA binding to SAM in **A.** reducing (10 mM TCEP) and **B.** oxidizing (10 mM GSSG; oxidized glutathione) conditions. No significant alteration in the dissociation constant (Kd) was evident.


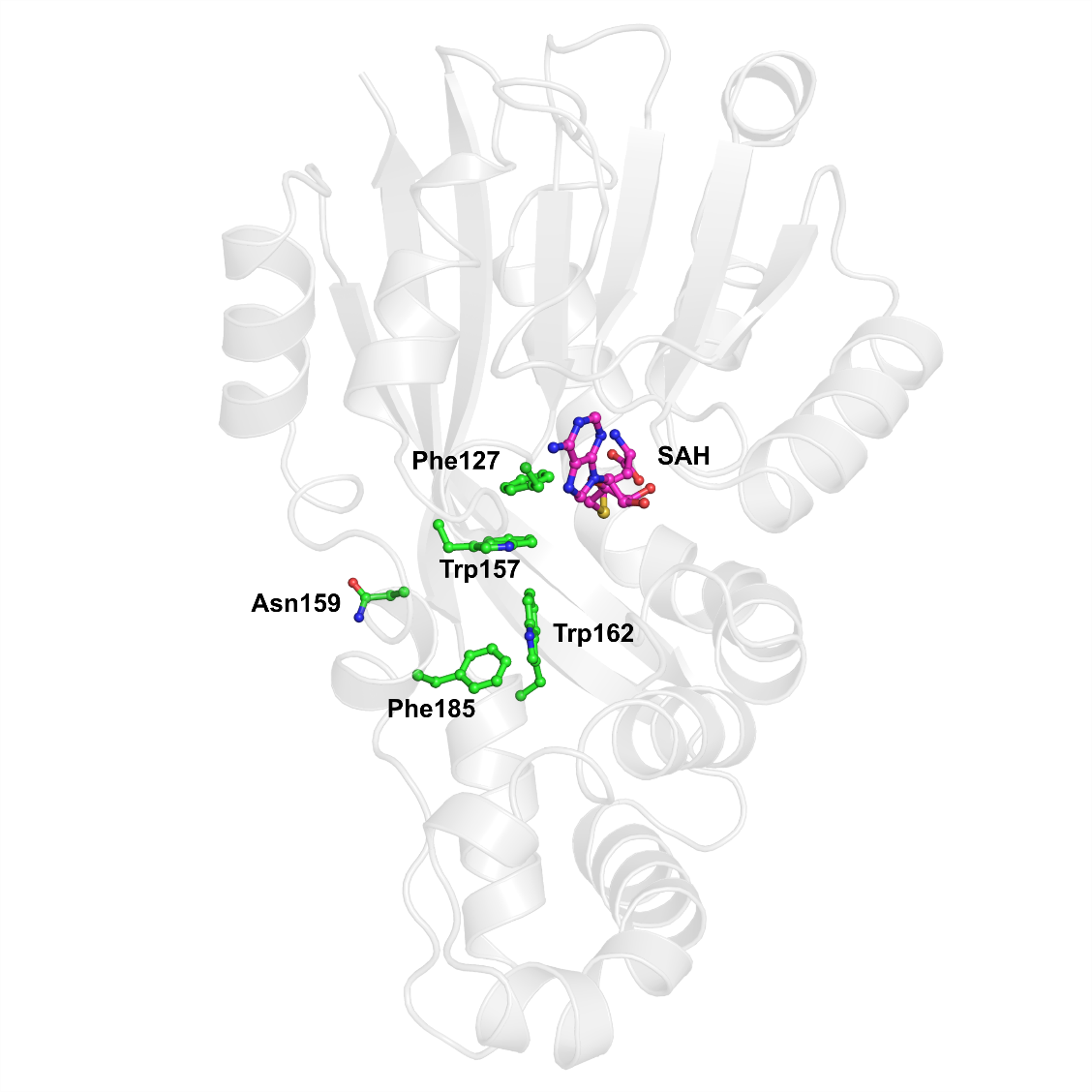


**Figure S4.** Location of residues chosen for mutagenesis in the GtmA-SAH complex.


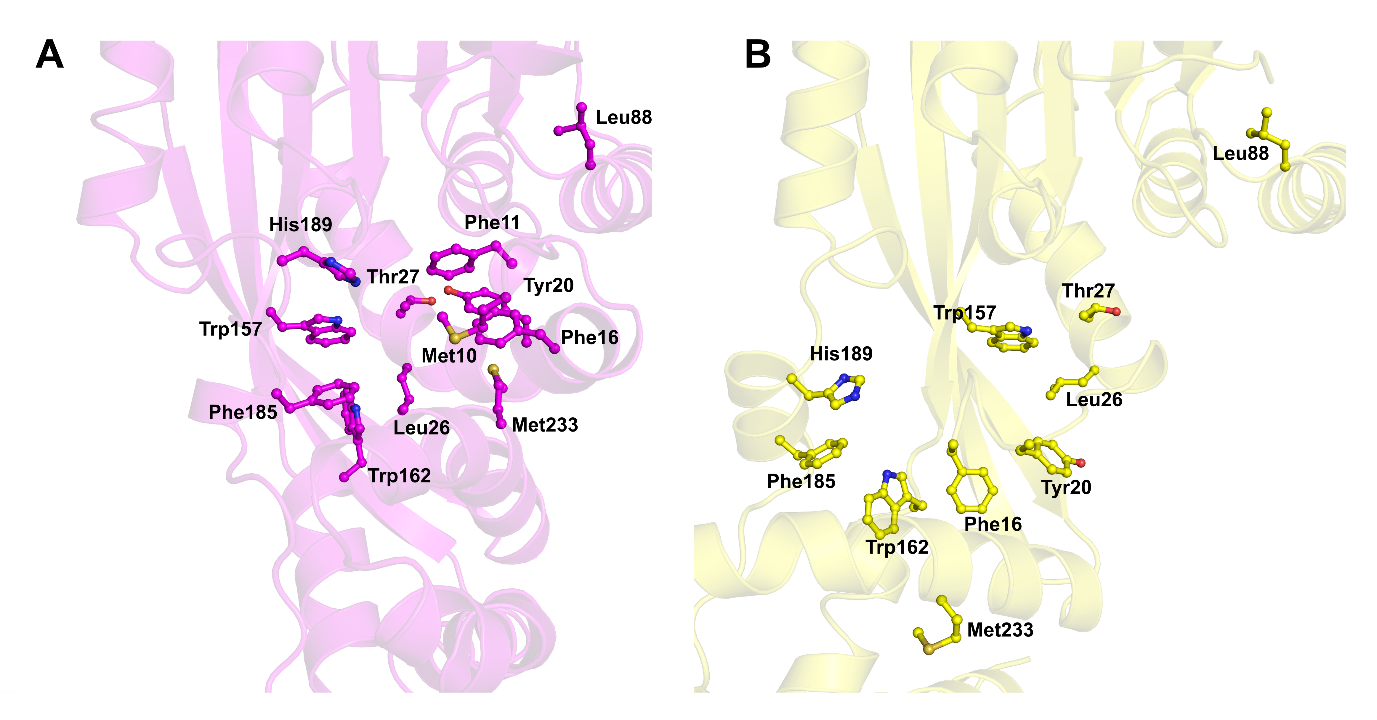


**Figure S5.** Disruption of the gliotoxin binding site in the SAM complex. Comparison of the **A.** SAH and **B.** SAM bound complex. Shown are the potential residues involved in gliotoxin binding, as determined by Duell *et al.* [6a]. Met10 and Phe11 are only present in the SAH structure and are not visible in the SAM complex.

**
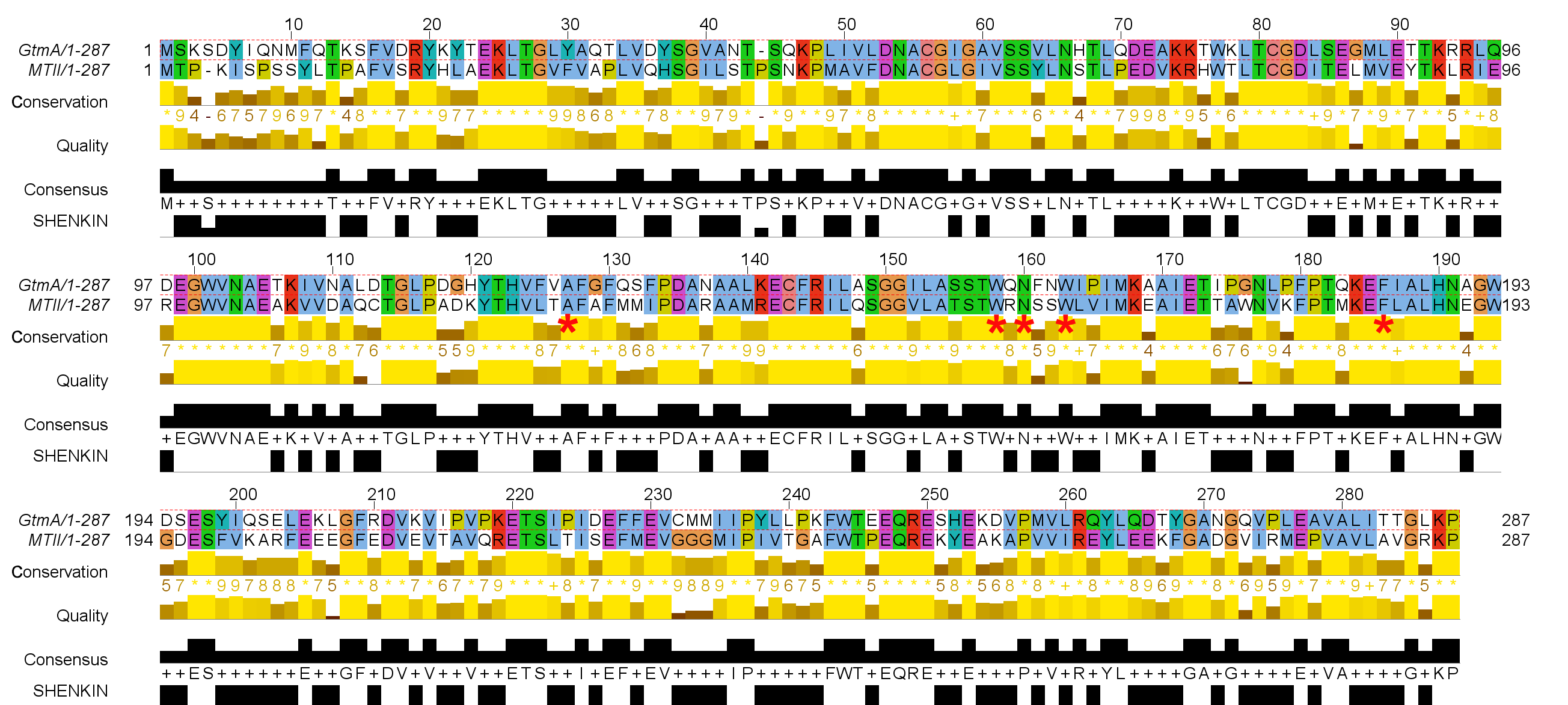
**

**Figure S6.** Clustal alignment of *A. fumigatus* GtmA amino acid sequence with *A. niger* MT-II. The red asterisks indicate conserved residues selected for mutagenesis. Residues in the alignment are colored according to the ClustalX shading model [7].

**Figure S7.** GtmA-catalyzed sequential formation of MmGT and BmGT over 60 min.

**
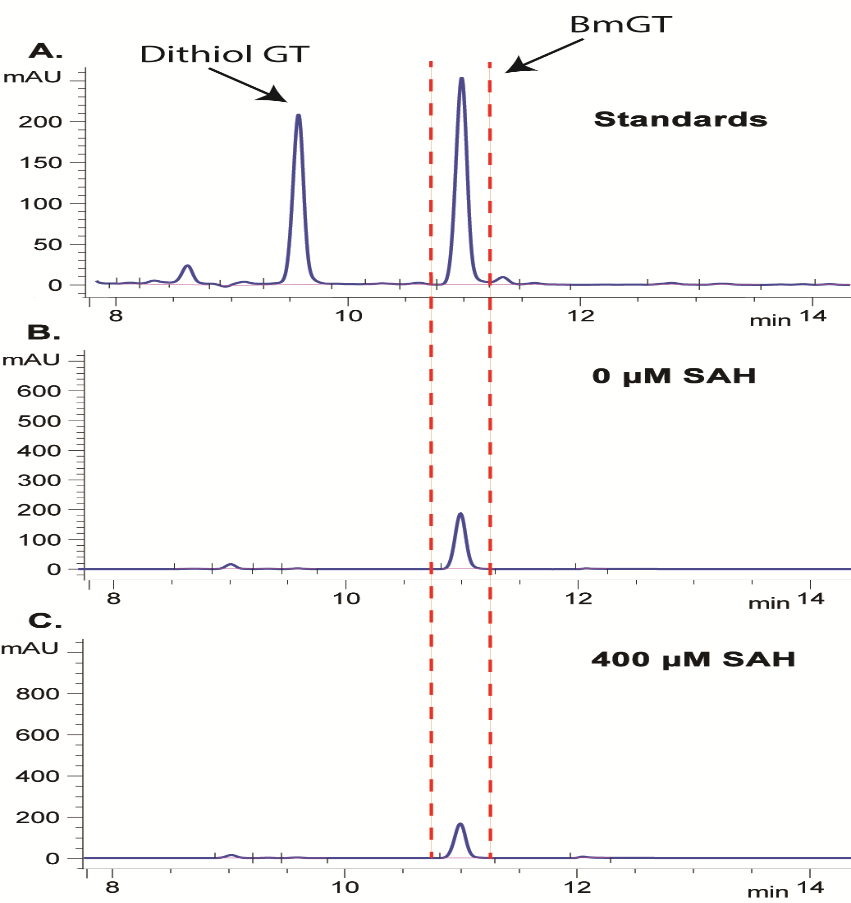
**

**Figure S8.** SAH does not significantly inhibit the activity of GtmA. **A.** RP-HPLC standards of dithiol gliotoxin and bis(methyl)gliotoxin showing the respective retention times. **B.** GtmA without added SAH. **C.** GtmA with SAH (400 µM) added to the reaction.

*
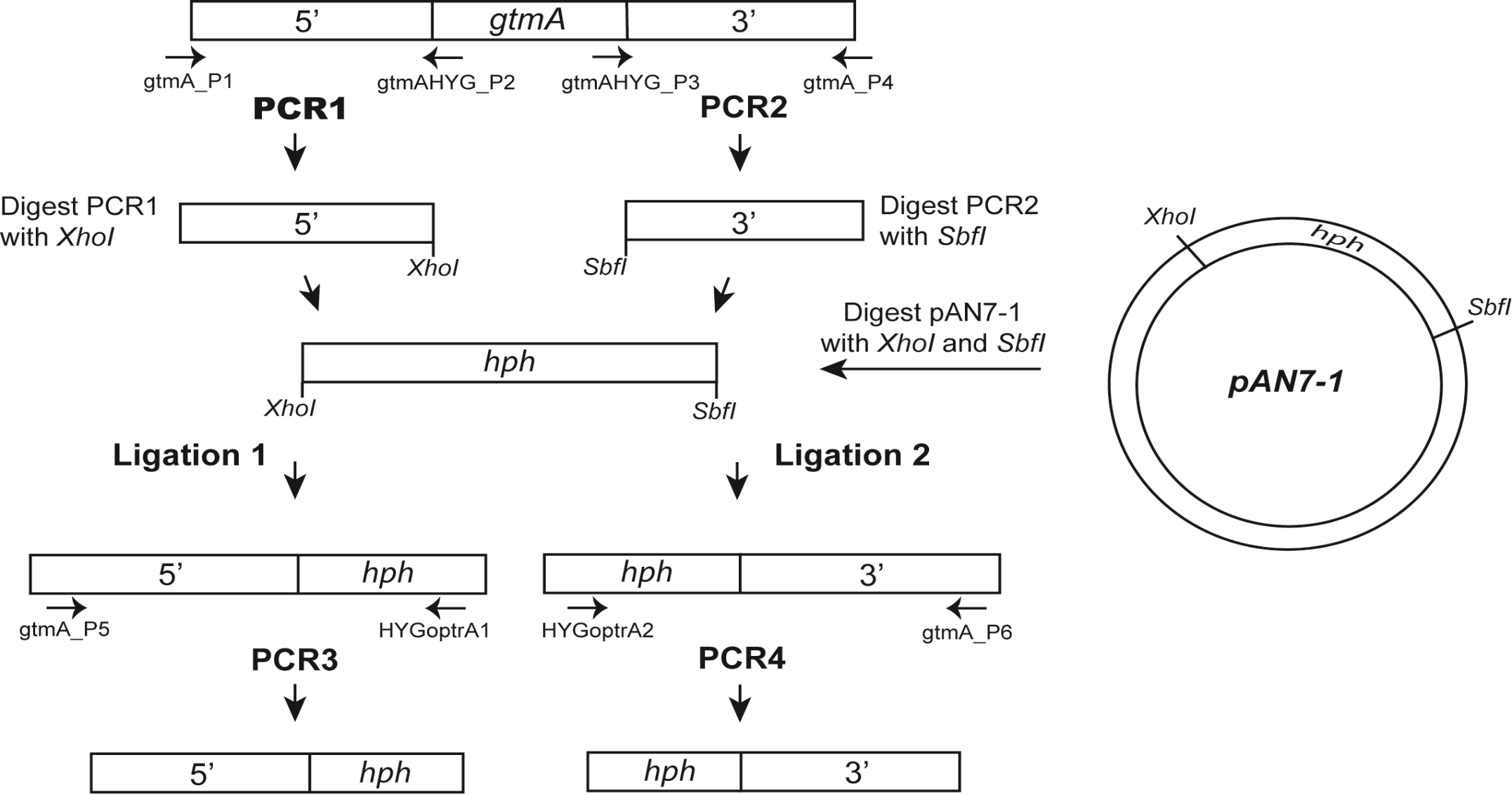
*

**Figure S9.** Schematic outlining the strategy used in the deletion of *gtmA* in *A. fumigatus* Δ*gliT* [4] and Δ*gliA* [6] using the hygromycin resistance marker.

**
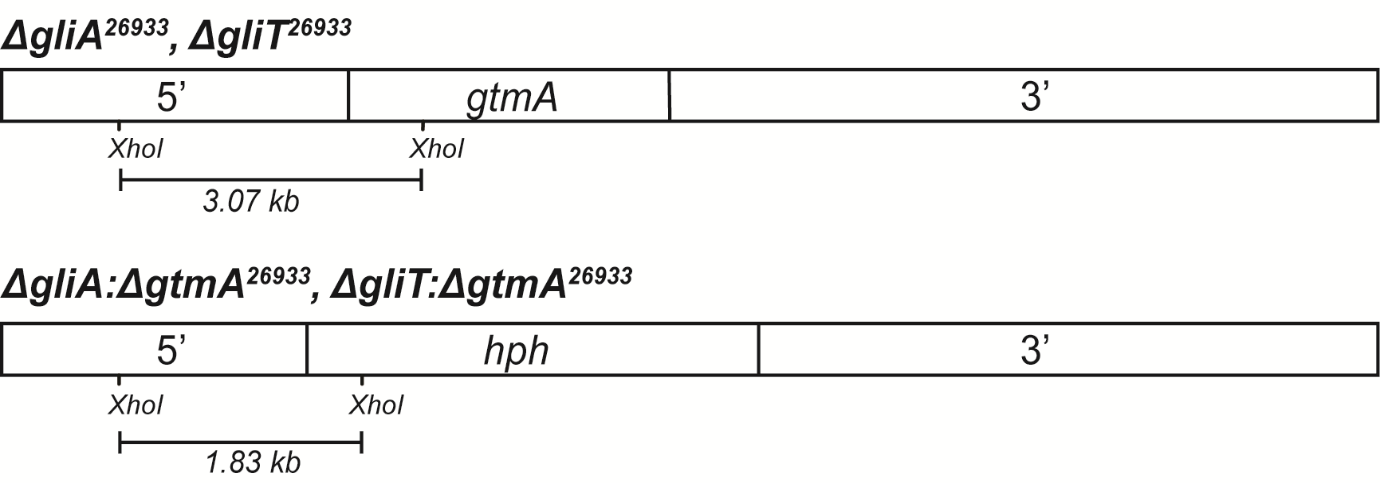
**

**Figure S10.** Schematic illustrating the Southern blot strategy employed for confirming the deletion of *gtmA* in Δ*gliT* and Δ*gliA*, respectively. The *XhoI* digested gDNA was screened using a 5’ probe. A correct gene deletion event should result in a band of 1.83 kb. The wild-type *Xho*I digested gDNA would be 3.07 kb.

**
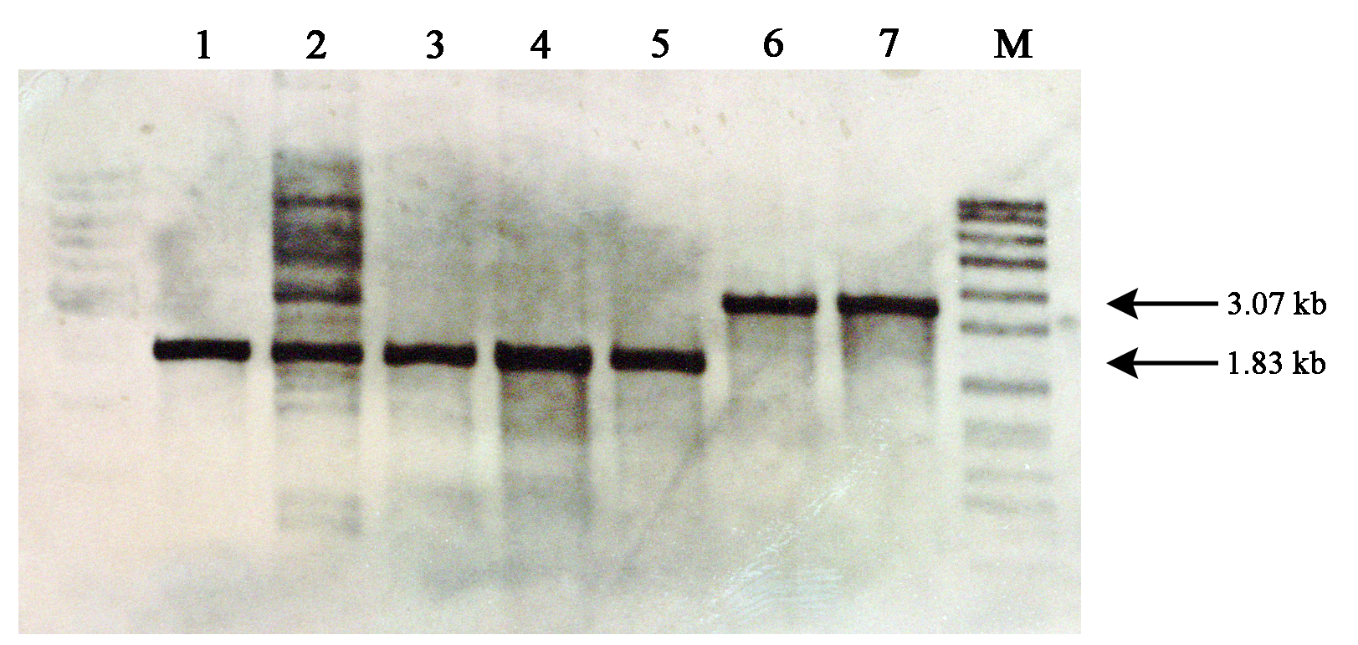
**

**Figure S11.** Identification of *A. fumigatus* Δ*gliT*::Δ*gtmA* and Δ*gliA*::Δ*gtmA* in second round Southern analysis of single spore isolates of Δ*gtmA-hph* transformants. Here, the 5’ DIG-labelled probe was used to detect the predicted presence of a 1.83 kb fragment in *Xho*I digested genomic DNA. Lane 1 - 3: Single spore isolates of potential Δ*gliT*::Δ*gtmA* mutant strains, Lanes 4-5; Single spore isolates of potential Δ*gliA*::Δ*gtmA* mutant strains, Lane 6; Δ*gliT* positive control (3.07 kb), Lane 7; Δ*gliA* positive control (3.07 kb).


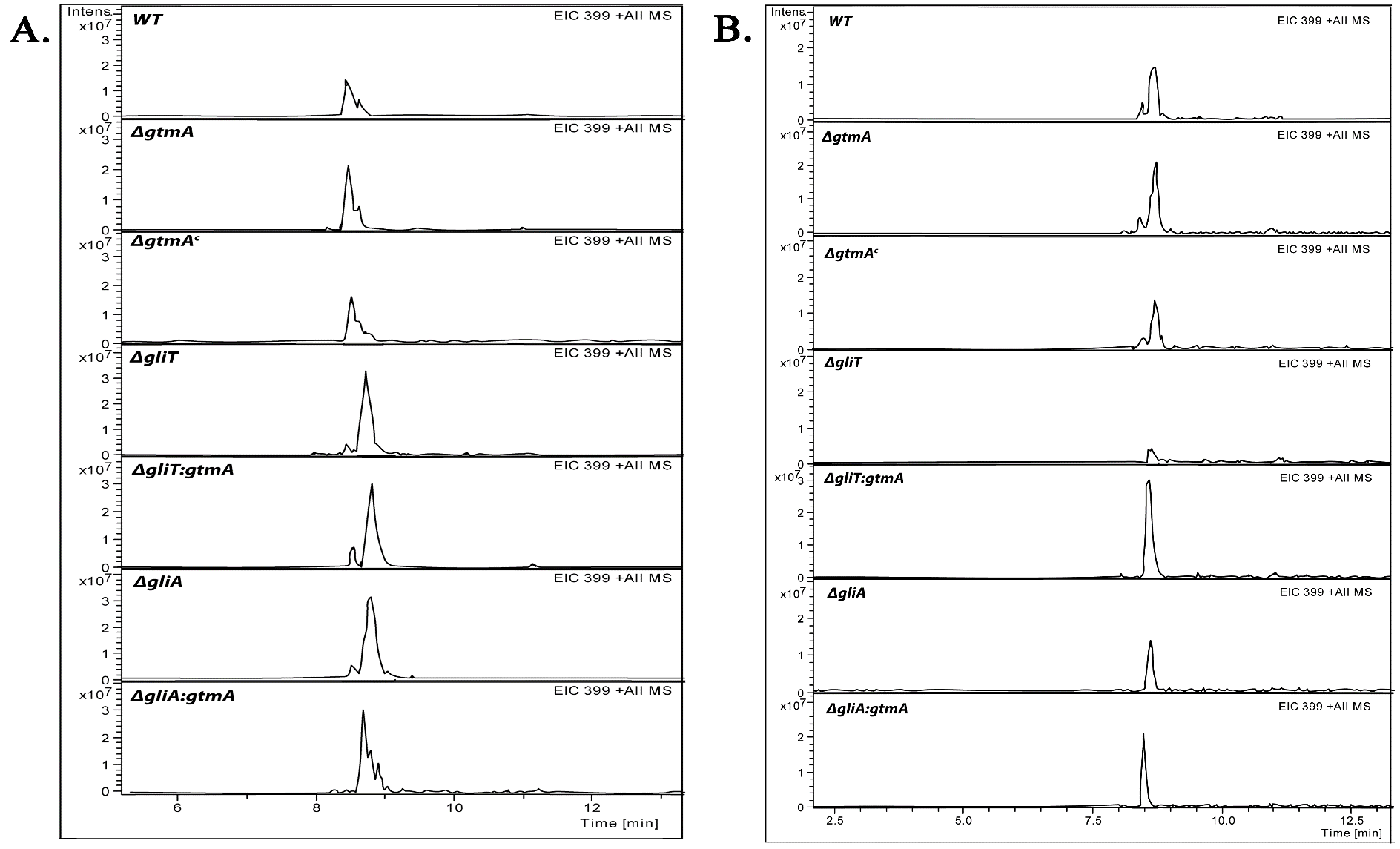


**Figure S12.** Corresponding chromatograms for SAM detection in *A. fumigatus* wild-type and selected mutants after 21 h growth in Czapeks Dox liquid media followed by 3 h of **A:** methanol exposure and **B:** Gliotoxin exposure.


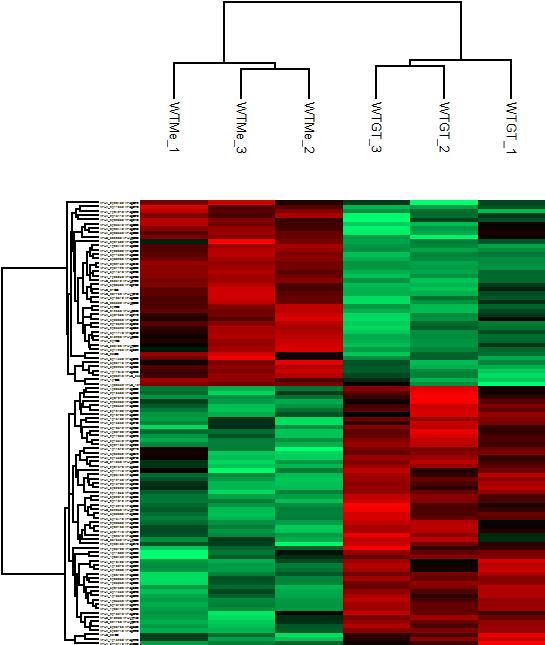


**Figure S13. A.** Heat map clustering of differentially expressed proteins in *A. fumigatus* wild-type exposed to MeOH vs GT identified by MS.


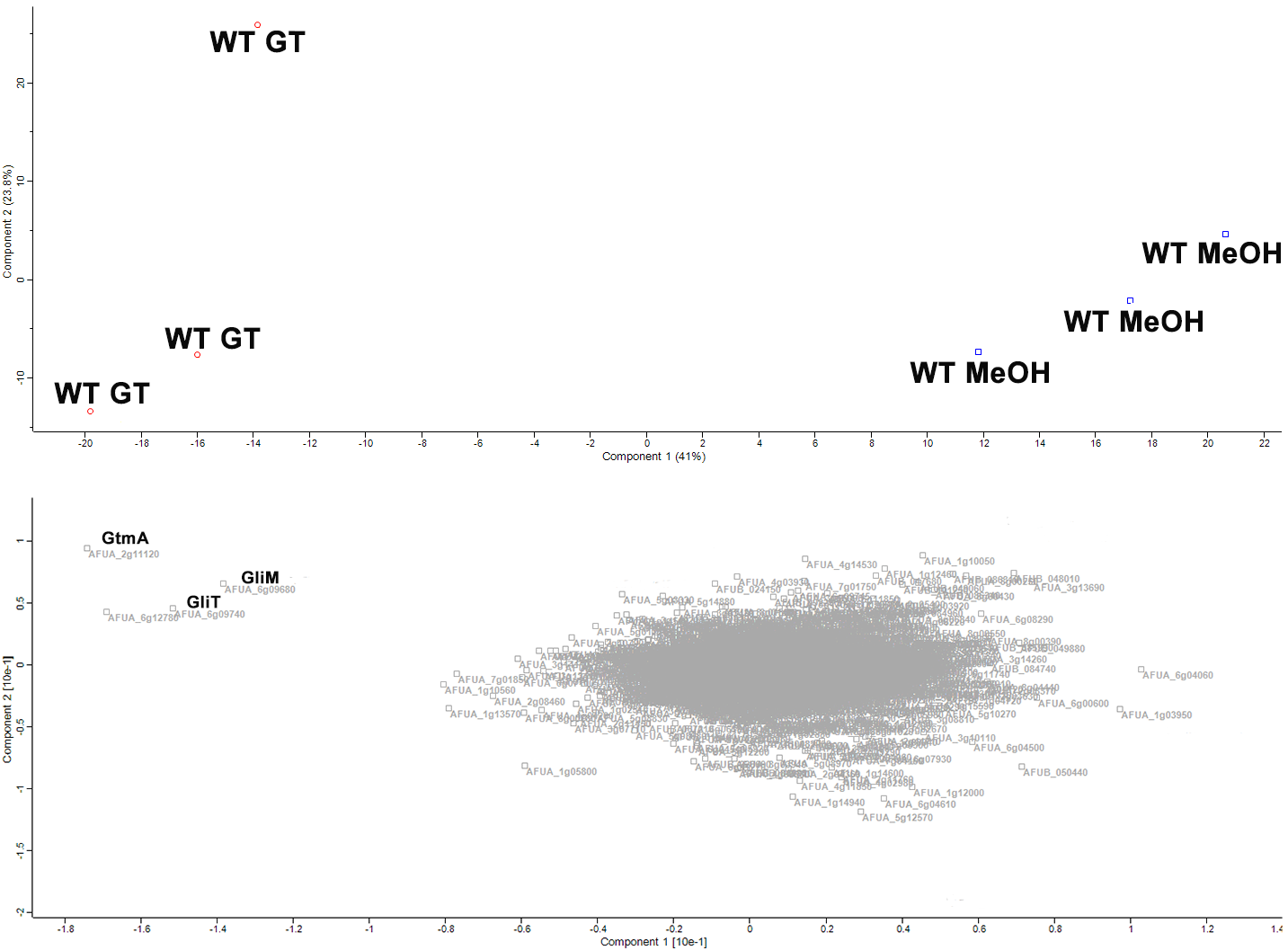


**Figure S13. B**. Principal component analysis (PCA) confirmation of the *A. fumigatus* wild-type MeOH vs GT treatment grouping.


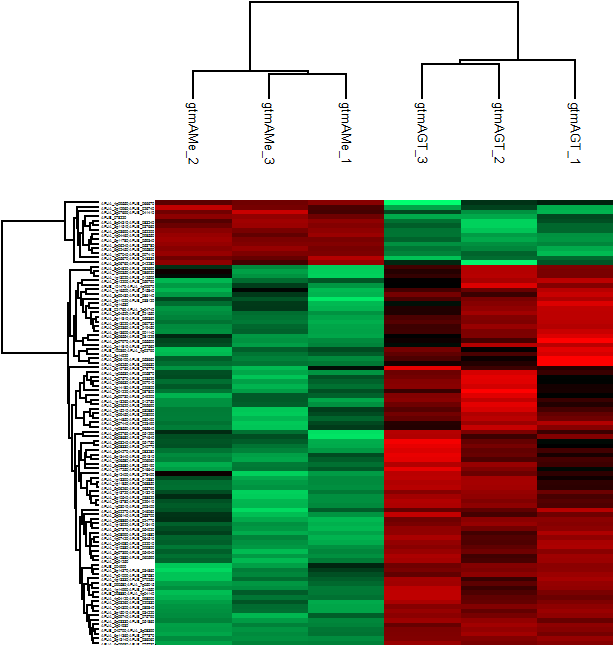


**Figure S14. A.** Heat map clustering of differentially expressed proteins in *A. fumigatus* Δg*tmA* exposed to MeOH vs GT identified by MS.


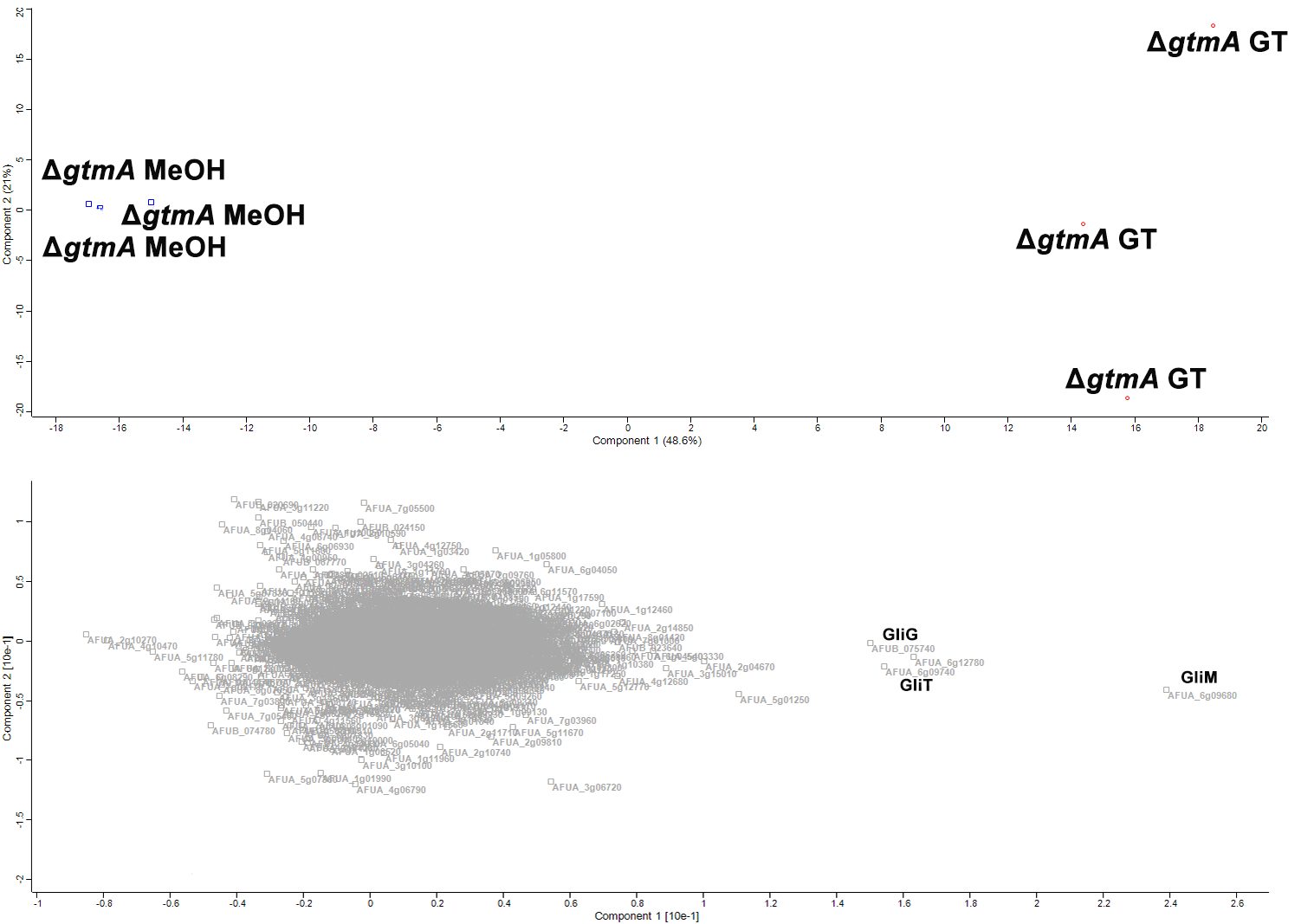


**Figure S14. B**. Principal component analysis (PCA) confirmation of the *A. fumigatus* Δg*tmA* MeOH vs GT treatment grouping.


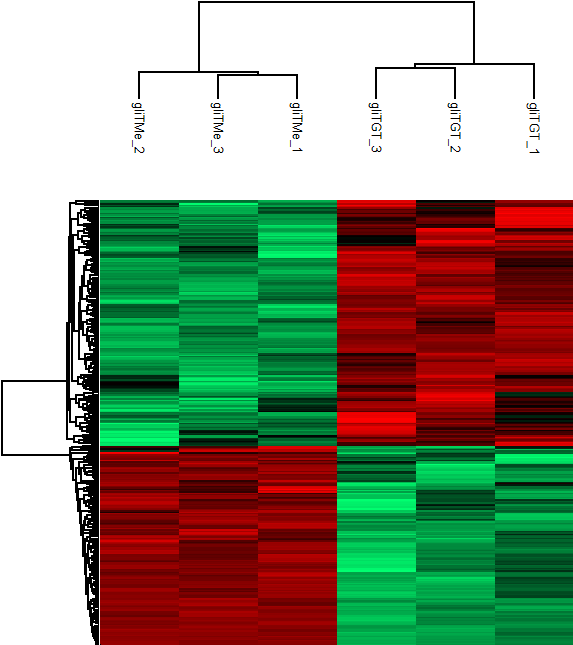


**Figure S15. A.** Heat map clustering of differentially expressed proteins in *A. fumigatus* Δg*liT* exposed to MeOH vs GT identified by MS.


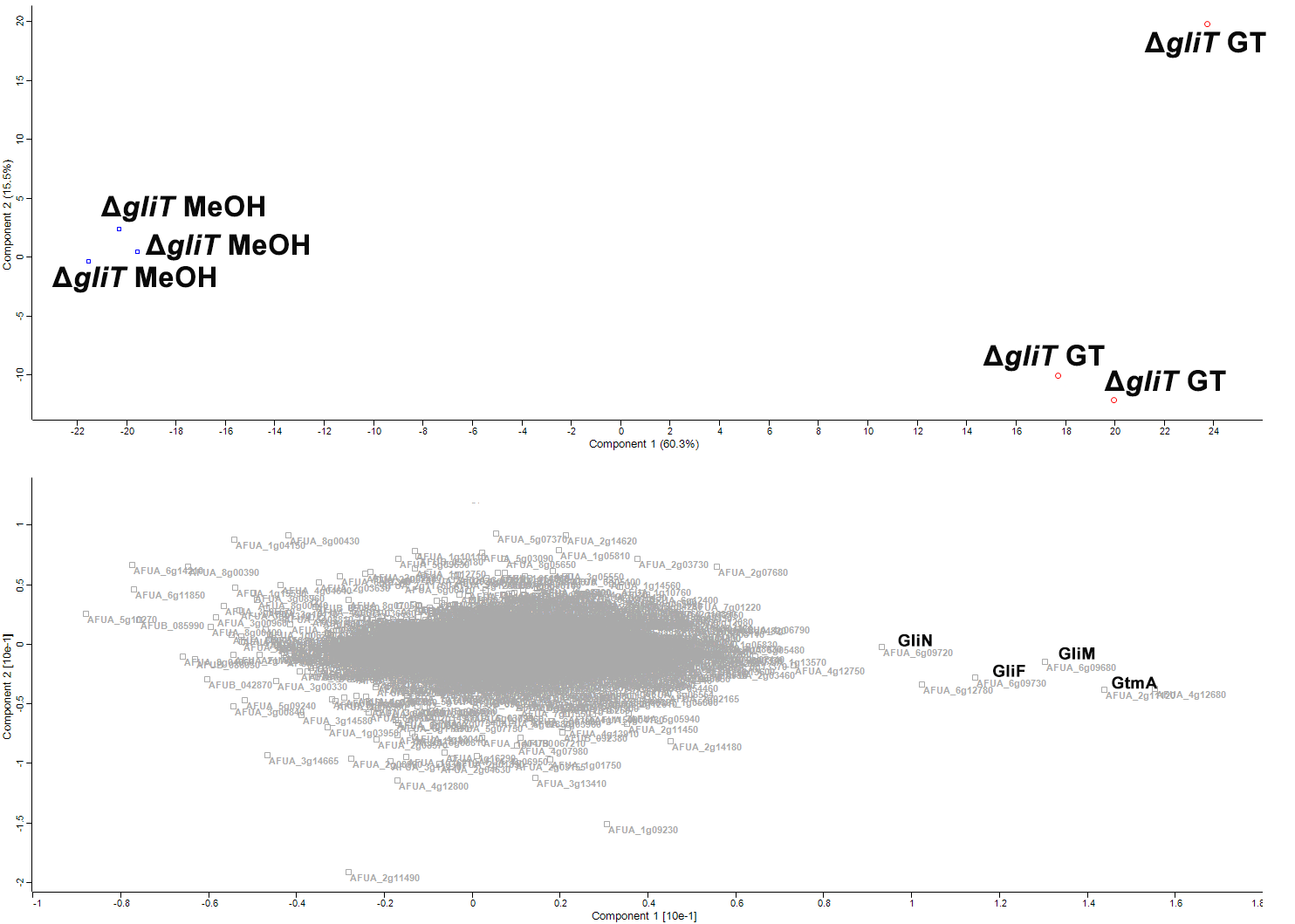


**Figure S15. B**. Principal component analysis (PCA) confirmation of the *A. fumigatus* Δ*gliT* MeOH vs GT treatment grouping.


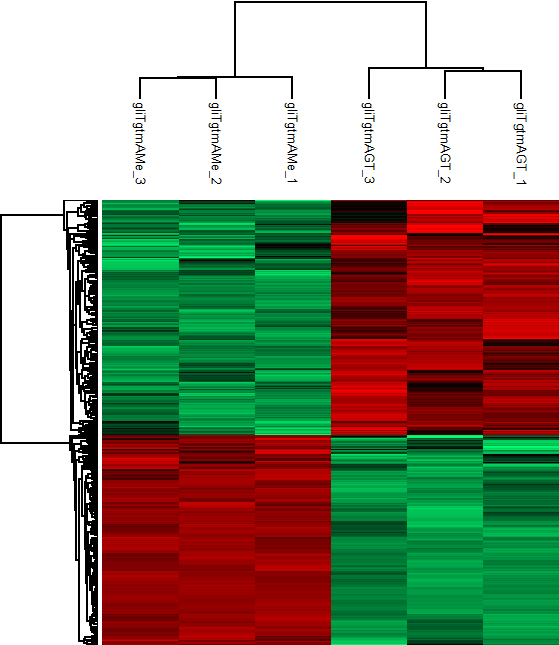


**Figure S16. A.** Heat map clustering of differentially expressed proteins in *A. fumigatus* Δ*gliT:gtmA* exposed to MeOH vs GT identified by MS.


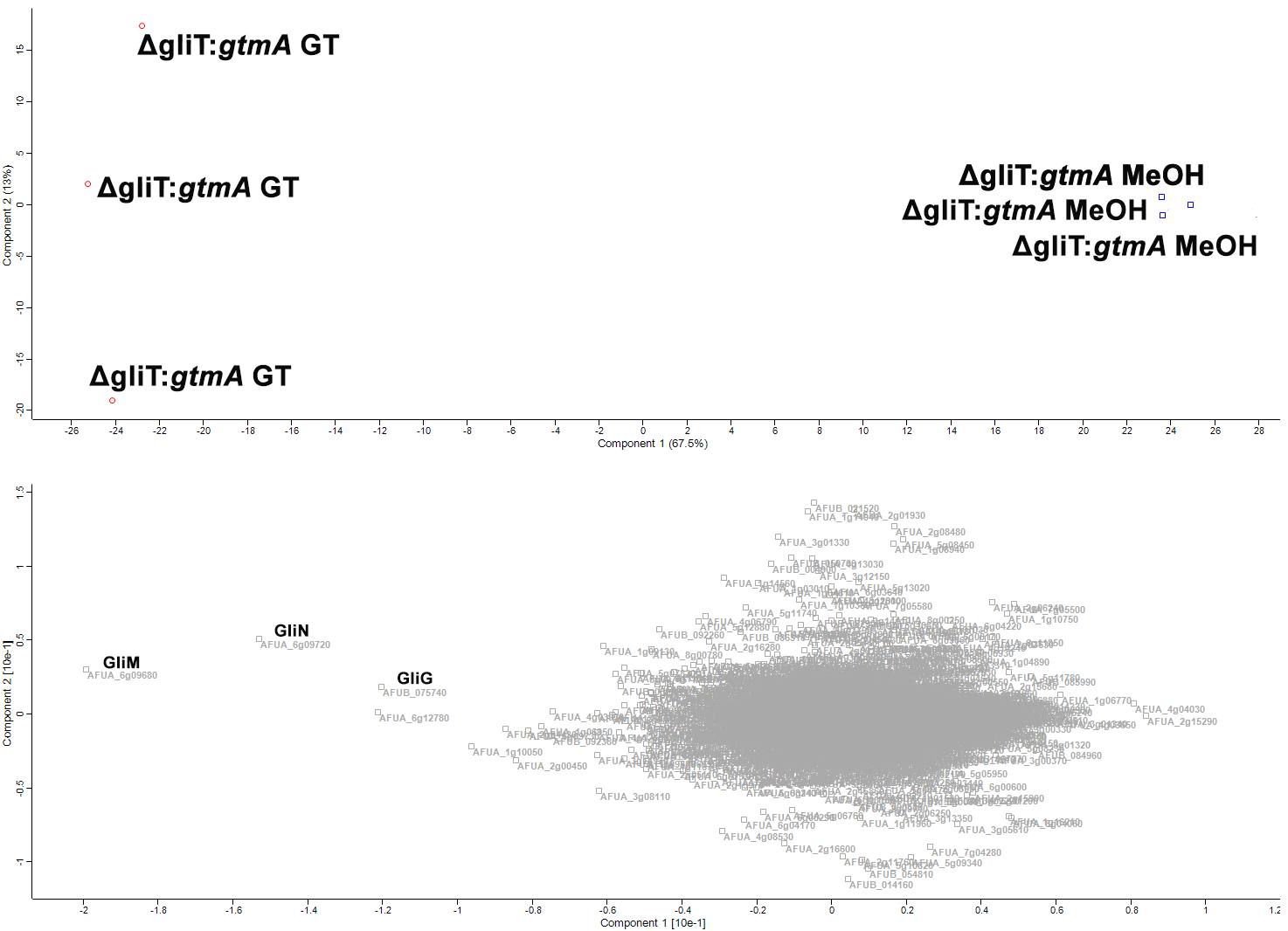


**Figure S16. B.** Principal component analysis (PCA) confirmation of the *A. fumigatus* Δ*gliT:gtmA* MeOH vs GT treatment grouping.


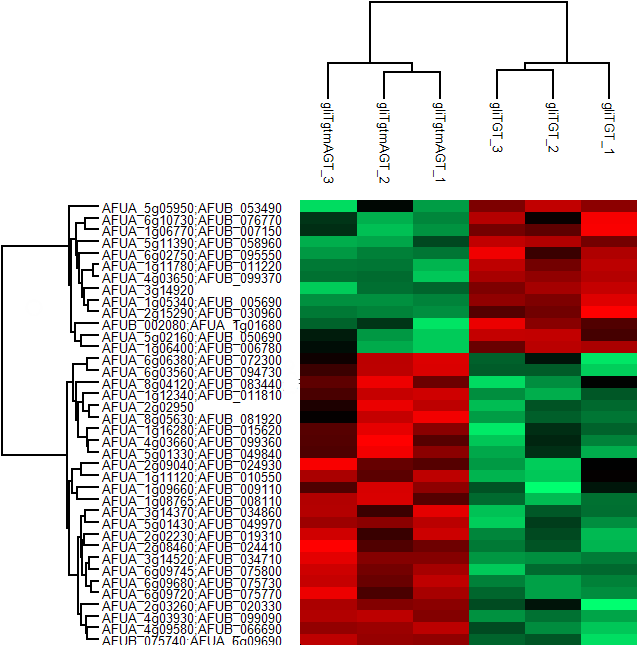


**Figure S17. A.** Heat map clustering of differentially expressed proteins in *A. fumigatus* Δ*gliT* vs. Δ*gliT:gtmA* exposed to GT identified by MS.


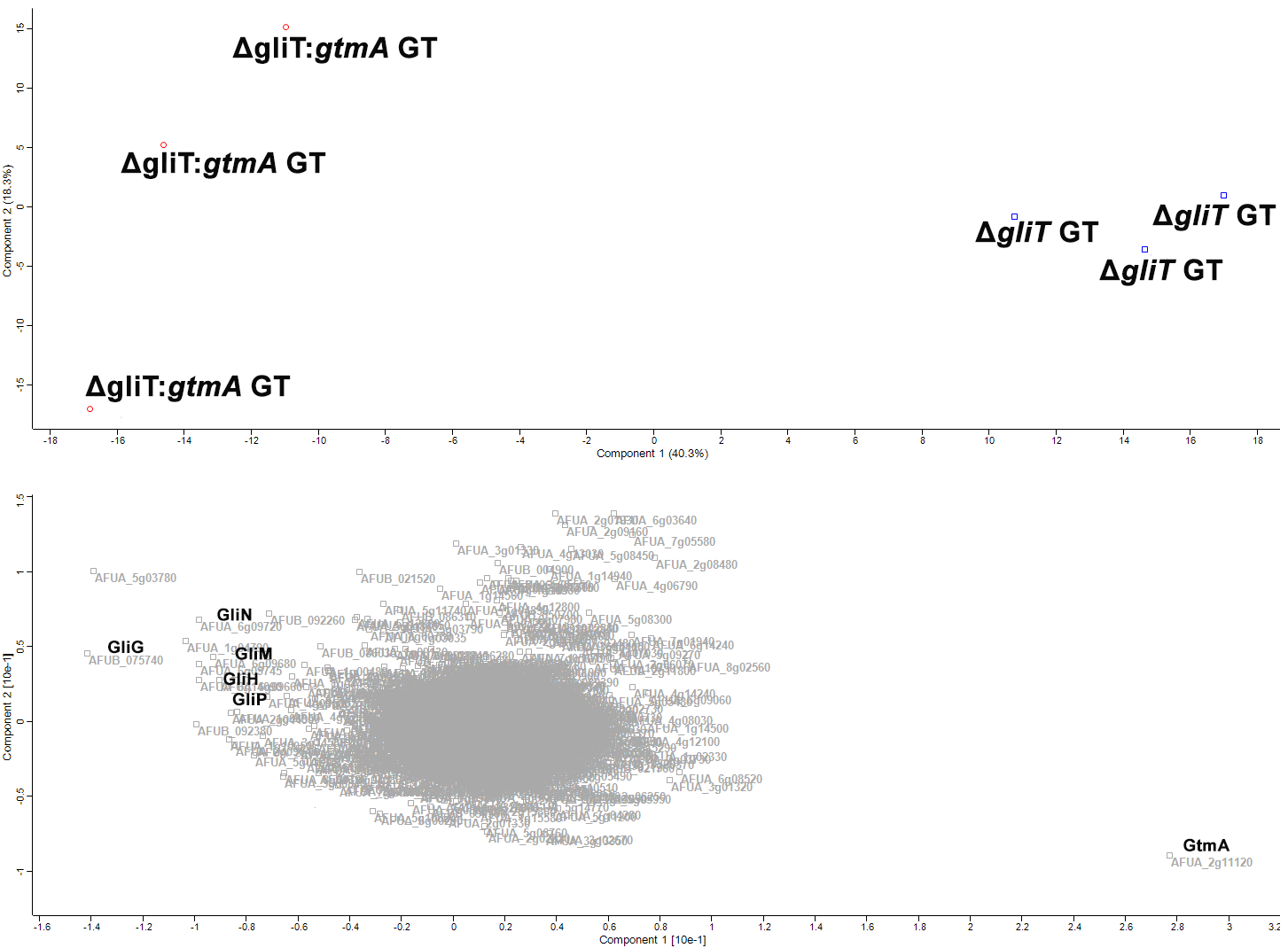


**Figure S17. B.** Principal component analysis (PCA) confirmation of the *A. fumigatus* Δ*gliT* vs. Δ*gliT:gtmA* exposed to GT treatment grouping.


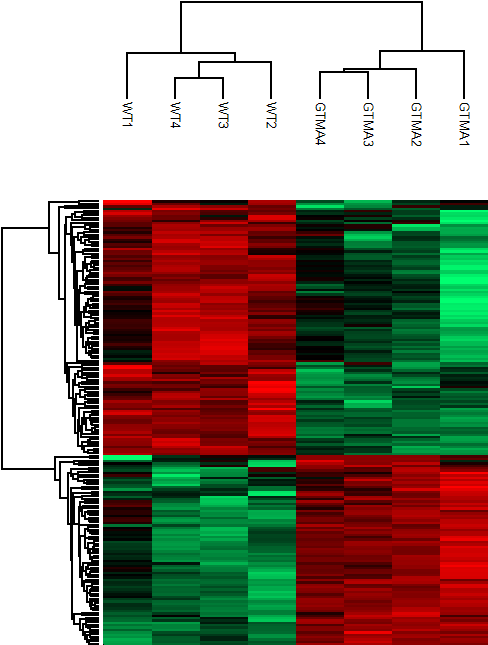


**Figure S18. A.** Heat map clustering of differentially expressed proteins in *A. fumigatus* wild-type vs. Δ*gtmA* identified by MS.


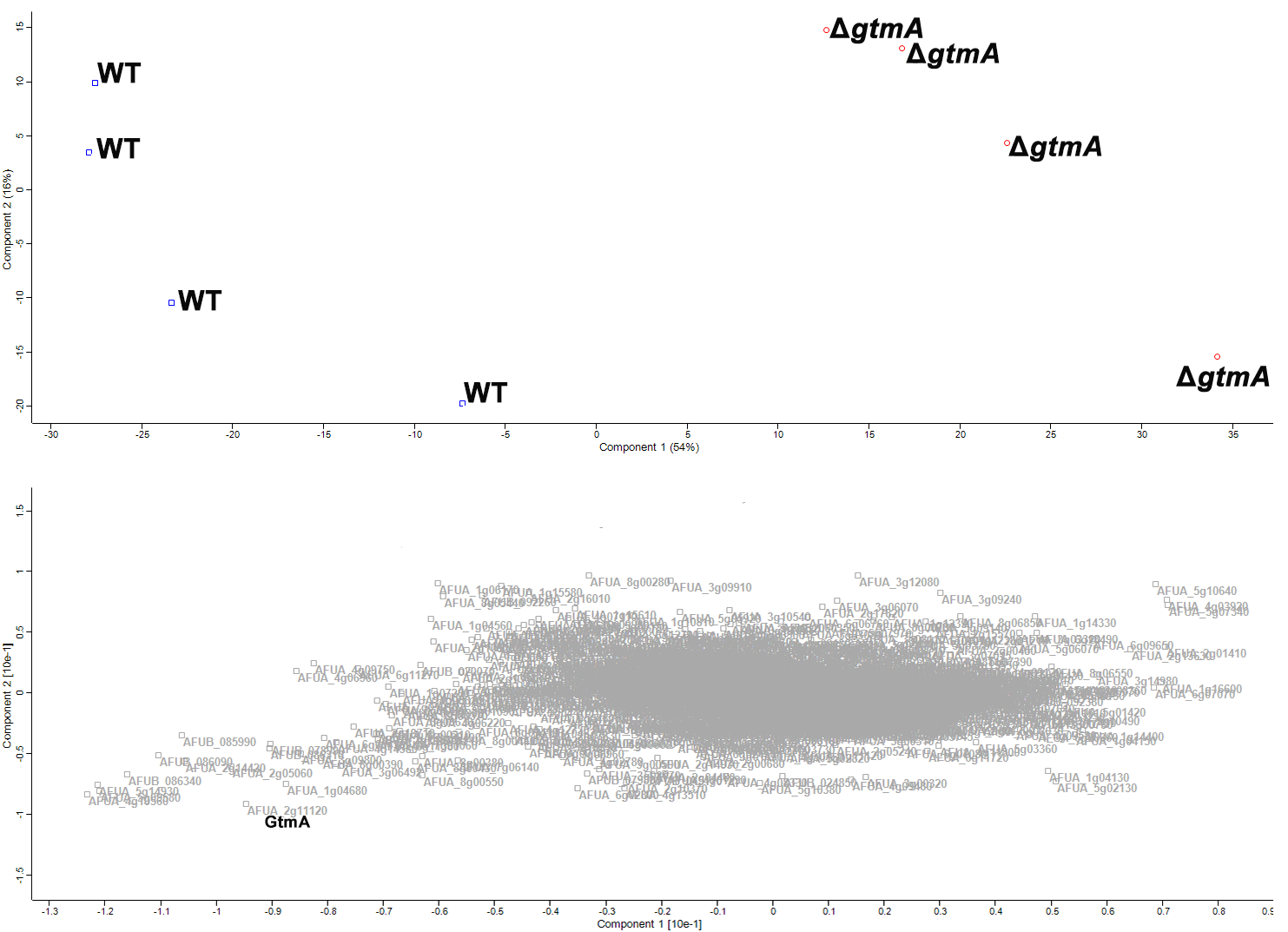


**Figure S18. B.** Principal component analysis (PCA) confirmation of the *A. fumigatus* wild-type vs. Δ*gtmA* identified by MS treatment grouping.


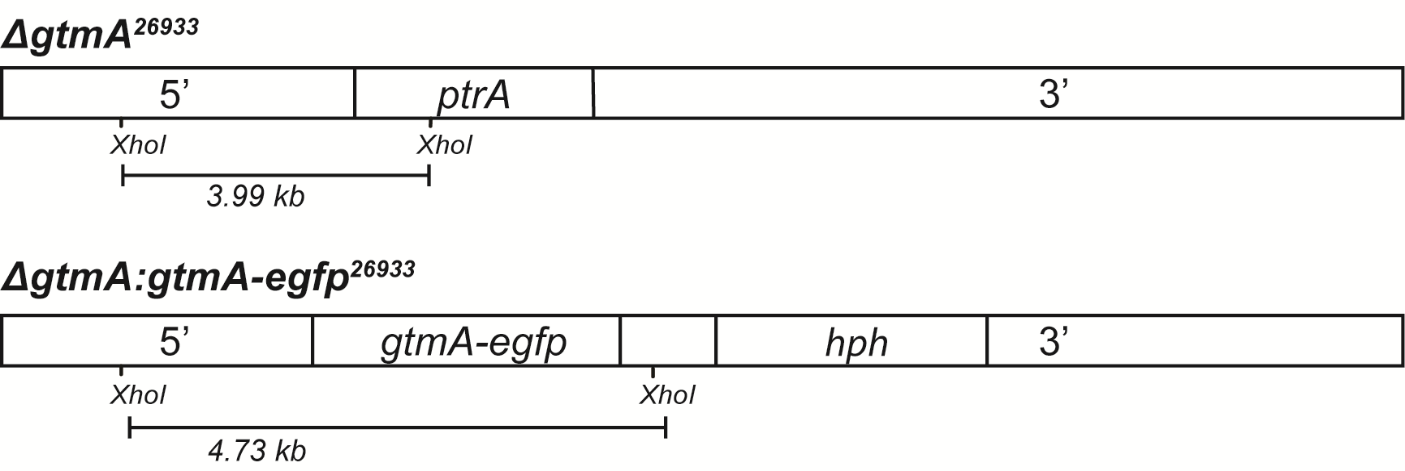


**Figure S19.** Schematic outlining the insertion of *gtmA-eGFP* into *A. fumigatus* Δ*gtmA*. A single homologous recombination between the 5’ flanking region results in the insertion of the construct into the 5’ flanking region of Δ*gtmA*, directly before the *ptrA* selection marker used to generate *A. fumigatus* Δ*gtmA: gtmA-eGFP* (4.73 kb).


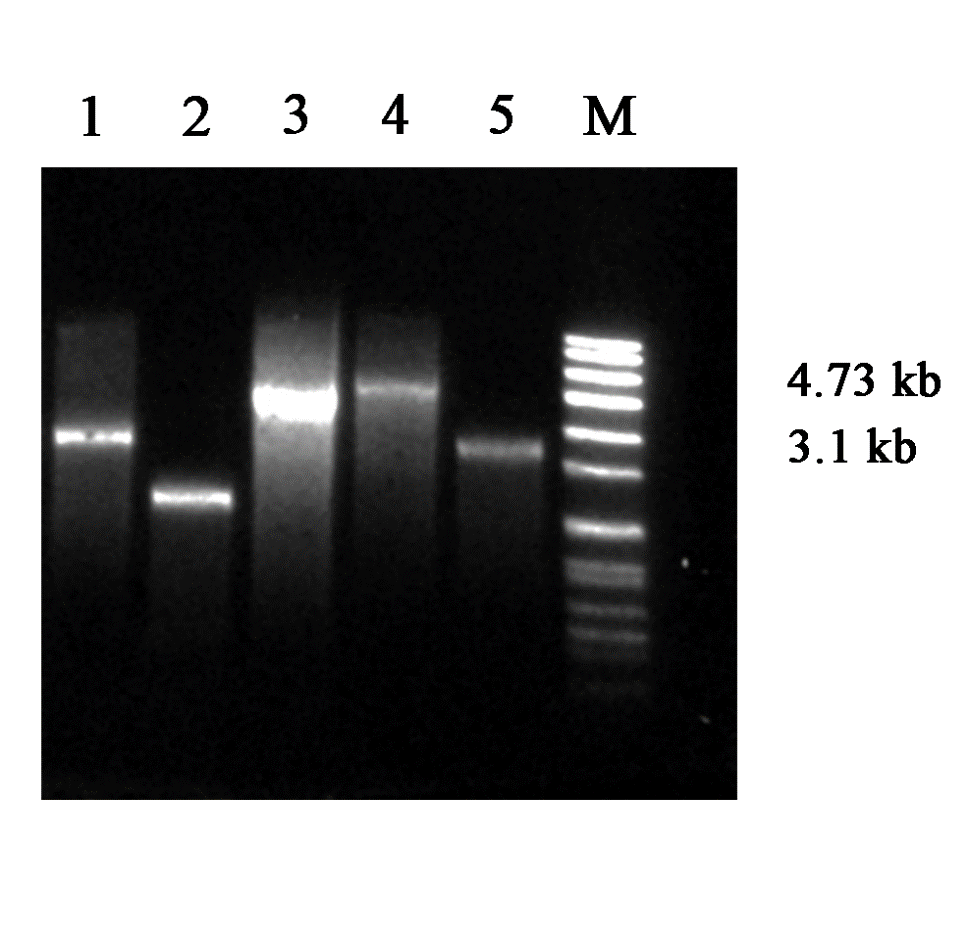


**Figure S20.** Identification of *A. fumigatus* Δ*gtmA: gtmA-eGFP* by Southern analysis. Second round Southern analysis of single spore isolates of Δ*gtmA*::*gtmA-eGFP* transformants. Here, the 5’ DIG-labelled probe was used to detect the predicted presence of a 4.73 kb fragment in *XhoI* digested genomic DNA. Lane 1 - 4: Single spore isolates of potential Δ*gtmA*::*gtmA-eGFP* transformants, Lane 5;Wild-type positive control


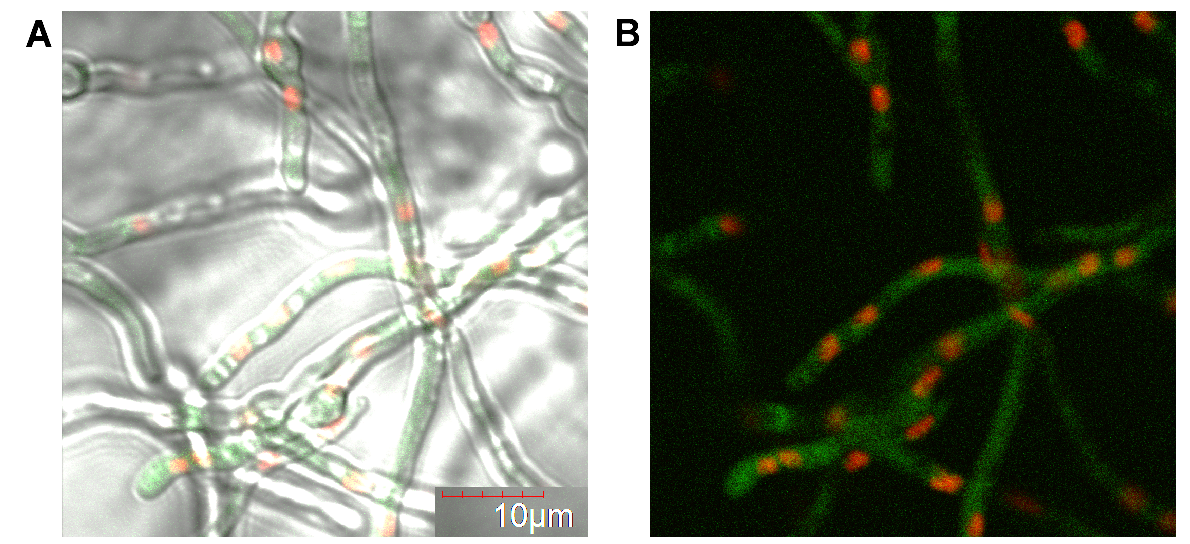


**Figure S21.** eGFP labelling of GtmA (GtmA::eGFP) revealed the rapid accumulation of this enzyme to the cytosol following gliotoxin exposure (5 µg/ml, 3 h). Histone 2A monomeric red fluorescent protein fusion (H2A::mRFP) visualizes the nuclei. The GtmA::eGFP signal was not detectable in the MeOH controls. **A**. Bright-field eGFP and mRFP signal overlay. **B**. eGFP and mRFP signal overlay.

**Supplemental Experimental Procedures.**

### Deletion of *gtmA* in the gliotoxin sensitive mutant backgrounds Δ*gliT* and Δ*gliA.*

The *gtmA* gene was also deleted in the gliotoxin sensitive mutant backgrounds Δ*gliT* and Δ*gliA*. As these gene deletions were also carried out in the ATCC26933 background, the results could be compared to the Δ*gtmA* single mutant. However, Δ*gliT* and Δ*gliA* were prepared using the pyrithiamine resistance gene *ptrA*. As such, the hygromycin resistance gene (*hph*) from the plasmid *pAN7-1* had to be used for the Δ*gliT*::Δ*gtmA* and Δ*gliA*::Δ*gtmA* mutant selections. As shown in Figure S9, *A. fumigatus* Δ*gliT* and Δ*gliA* were co-transformed with two DNA constructs, each of which contained an incomplete fragment of *hph*. These fragments were fused to 1.1 kb and 1.1 kb of *gtmA* flanking sequences. These marker fragments shared a 572 bp overlap within the *hph* cassette which served as a potential recombination site during transformation. Throughout transformation, homologous integration of each fragment into the genome flanking *gtmA* allowed the recombination of the *hph* fragments and the generation of an intact resistance gene at the site of recombination.

Two rounds of PCR generated each fragment. Initially, each flanking region was amplified from ATCC26933 genomic DNA using the primers *gtmA*-P1and *gtmA_HYG*-P2 for flanking region A (1.15 kb) and *gtmA_HYG*-P3 and *gtmA*-P4 for flanking region B (1.10 kb). Following gel-purification the fragments were digested with *Xho*I and *Sbf*I, respectively. The *hph* selection marker was released from plasmid *pAN7-1*by digestion with *Xho*I and *Sbf*I and ligated with the two flanking regions, A and B. Two overlapping fragments were amplified from the ligation product using primers *gtmA*-P5 and PTRoptrA2 for fragment C (2.32 kb), and primers *gtmA*-P6 and PTRoptrA1 for fragment D (2.76 kb). The PCR products were gel-purified to remove any non-specific products and then concentrated prior to protoplast transformation. Finally, Δ*gliT* and Δ*gliA* were independently transformed in concert with the two overlapping fragments.

**Transformation and selection for deletion of *gtmA* from *A. fumigatus* ATCC26933 Δ*gliT* and Δ*gliA***

Approximately 3 μg of both 5’ and 3’ construct were used per transformation event. The resultant transformants were selected on agar plates containing hygromycin (100 μg/ml). The colonies that grew on these were considered resistant and, therefore, predicted to have reconstituted *hph* incorporated into the genome.

For the transformation event, 12 Δ*gliT* background and 9 Δ*gliA* background colonies were observed on hygromycin selection plates. Genomic DNA from these transformants was screened by Southern analysis using a 5’ probe. Figure S8 illustrates the Southern strategy employed to confirm Δ*gliT*::Δ*gtmA* and Δ*gliA*::Δ*gtmA* deletions. The mutant band (*ΔgtmA*; 1.83 kb) was present in two Δ*gliT*::Δ*gtmA* transformants and one Δ*gliA*::Δ*gtmA* transformant. These three colonies were selected for single spore isolation. Single spore isolates of Δ*gliT*::Δ*gtmA* and Δ*gliA*::Δ*gtmA* were digested with *Xho*I and a second round of Southern blot analysis using the 5’ probe was performed. This confirmed the single integration of the *ΔgtmA* deletion cassette at the correct locus (Figure S11).

### Generation of *gtmA-eGFP* Gene Fusion Construct

The *pUCGH* eGFP vector containing *hph* [8] under the control of a *Potef* constitutive promoter was digested with the enzymes *Kpn*I and *Xma*I. This released the *Potef* promoter from the vector and facilitated the cloning of *gtmA* under the control of its native promoter into the *pUCGH* vector. The *gtmA* gene with both 1 kb upstream and the stop codon removed was amplified from *A. fumigatus* 26933 DNA (2036 bp) using the primers *gtmA*_GFP_F and *gtmA*_GFP_R. This construct was then double digested with *Kpn*I and *Xma*I, gel purified and, finally, ligated to the digested *pUCGH* vector. The final vector (*pUCGHgtmA*) was linearized with *Kpn*I and transformed into *A. fumigatus* Δ*gtmA*. This strategy targeted the *gtmA-egfp-hph* construct to the original *gtmA* locus of the Δ*gtmA* mutant. 6 µg of the linearised construct was transformed into Δ*gtmA* protoplasts.

### Southern blot Analysis of *A. fumigatus gtmA-eGFP* Transformants

Figure S14 illustrates the Southern strategy employed to confirm *gtmA-eGFP* insertion into Δ*gtmA*. Genomic DNA from all four potential transformants was digested with *XhoI* and probed with the 5’ probe. Southern blot analysis identified a single transformant with *gtmA-GFP* insertion at the correct locus (Colony 4). This transformant had the predicted fragment size of 4.73 kb when analysed by Southern blot

### Transformation of *A. fumigatus* Δ*gtmA*::*gtmA-eGFP* with a Histone 2A monomeric red fluorescent protein fusion (H2A::mRFP) plasmid

In order to visualise the nuclei of Δ*gtmA:gtmA-eGFP* during confocal microscopy, a Histone 2A monomeric red fluorescent protein fusion (H2A::mRFP) plasmid [9] was linearised using *EcoRI* (5 µg) and transformed into *ΔgtmA:gtmA-eGFP* protoplasts. The resultant transformants were selected for on agar plates containing phleomycin (100 μg/ml) at 30 °C and screened for mRFP expression by confocal microscopy.

**MmGT preparation for GtmA Km evaluation**

Prior to analysis, purified MmGT was prepared as follows: Reactions were set up with 750 µM dithiol gliotoxin, 1 µM GtmA, 1.5 mM SAM and incubated at 37 °C for 30 min. Reactions were stopped by addition of TCA to 15 % and centrifuged to remove precipitated protein. Ethyl acetate was used to extract gliotoxin metabolites using a 1:1 volume and 2 consecutive extractions. Ethyl acetate extracts were dried to completion, resuspended in minimal volume methanol and purified by RP-HPLC fractionation. Note: purified MmGT fraction contained no dithiol gliotoxin.

**Supplemental References.**

1. Winn, M. D. et al. 2011 Overview of the CCP4 suite and current developments. *Acta Crystallogr. D. Biol. Crystallogr.* **67**, 235–42. (doi:10.1107/S0907444910045749)

2. Weiss, M. S. 2001 Global indicators of X-ray data quality. *J. Appl. Crystallogr.* **34**, 130–135. (doi:10.1107/S0021889800018227)

3. Chen, V. B., Arendall, W. B., Headd, J. J., Keedy, D. A., Immormino, R. M., Kapral, G. J., Murray, L. W., Richardson, J. S. & Richardson, D. C. 2010 MolProbity: all-atom structure validation for macromolecular crystallography. *Acta Crystallogr. Sect. D-Biological Crystallogr.* **66**, 12–21. (doi:10.1107/S0907444909042073)

4. Schrettl, M., Carberry, S. & Kavanagh, K. 2010 Self-protection against gliotoxin—a component of the gliotoxin biosynthetic cluster, GliT, completely protects Aspergillus fumigatus against exogenous. *PLoS Pathog.* **6**, e1000952. (doi:10.1371/journal.ppat.1000952)

5. Dolan, S.K., Owens, R.A., O’Keeffe, G., Hammel, S., Fitzpatrick, D.A., Jones, G.W.,

and Doyle, S. (2014). Regulation of nonribosomal peptide synthesis: bis-

thiomethylation attenuates gliotoxin biosynthesis in Aspergillus fumigatus. *Chem.*

*Biol*. **21**, 999–12.

6. Owens, R. A. et al. 2015 Interplay between Gliotoxin Resistance, Secretion, and the Methyl/Methionine Cycle in Aspergillus fumigatus. *Eukaryot. Cell* **14**, 941–57. (doi:10.1128/EC.00055-15)

6a. Duell, E. R., Glaser, M., Le Chapelain, C., Antes, I., Groll, M. & Huber, E. M. 2016 Sequential inactivation of gliotoxin by the S-methyltransferase TmtA. *ACS Chem. Biol.* , acschembio.5b00905. (doi:10.1021/acschembio.5b00905)

7. Waterhouse, A. M., Procter, J. B., Martin, D. M. A., Clamp, M. & Barton, G. J. 2009 Jalview Version 2--a multiple sequence alignment editor and analysis workbench. *Bioinformatics* **25**, 1189–91. (doi:10.1093/bioinformatics/btp033)

8. Langfelder, K., Philippe, B. & Jahn, B. 2001 Differential expression of the Aspergillus fumigatus pksP gene detected in vitro and in vivo with green fluorescent protein. *Infect Immun* **69**, 6411-18*.* (doi:10.1128/IAI.69.10.6411)

9. Sarikaya Bayram, O. et al. 2010 LaeA control of velvet family regulatory proteins for light-dependent development and fungal cell-type specificity. *PLoS Genet.* **6**, e1001226. (doi:10.1371/journal.pgen.1001226)
